# Reversal of ACLF and ALF using whole blood extracorporeal system combining HLA-depleted liver organoids with granulocyte-monocyte apheresis

**DOI:** 10.1016/j.jhep.2025.08.038

**Published:** 2025-10-02

**Authors:** Hitomi Yamaguchi, Yosuke Yoneyama, Kentaro Ichimura, Kanae Ohtsu, Mika Soen, Chiharu Moriya, Maki Kumagai, Robert P. Myers, G. Mani Subramanian, Takanori Takebe

**Affiliations:** 1 Department of Organogenesis and Neogenesis, Graduate School of Medical and Dental Sciences, Institute of Science Tokyo (Science Tokyo), 1-5-45, Yushima, Bunkyo-ku, Tokyo 113-8510, Japan; 2 Human Biology Research Unit, Institute of Integrated Research, Institute of Science Tokyo (Science Tokyo), 1-5-45, Yushima, Bunkyo-ku, Tokyo 113-8510, Japan; 3 Department of Stem Cell and Organoid Medicine, Graduate School of Medicine, The University of Osaka, Suita, Osaka 565-0871, Japan; 4 Department of Gastroenterological Surgery I, Hokkaido University Graduate School of Medicine, Kita 15, Nishi 7, Kita-ku, Sapporo, 060-8638, Japan; 5 Kanzo Biomedicines, Inc., 470 Noor Avenue, Ste B#1114, South San Francisco, CA 94080, USA; 6 Premium Research Institute for Human Metaverse Medicine (WPI-PRIMe), The University of Osaka, Suita, Osaka 565-0871, Japan; 7 Division of Gastroenterology, Hepatology and Nutrition & Division of Developmental Biology, Cincinnati Children’s Hospital Medical Center, 3333 Burnet Avenue, Cincinnati, OH 45229-3039, USA; 8 Center for Stem Cell and Organoid Medicine (CuSTOM), Cincinnati Children’s Hospital Medical Center, 3333 Burnet Avenue, Cincinnati, OH 45229-3039, USA; 9 Department of Pediatrics, University of Cincinnati College of Medicine, 3333 Burnet Avenue, Cincinnati, OH 45229-3039, USA

**Keywords:** Immune-evasive iPSC therapy, whole blood extracorporeal circulation, systemic inflammation resolution, hepatocyte nuclear factor 4 alpha (HNF4α), alginate-encapsulation, liver regeneration

## Abstract

**Background & Aims:**

Acute-on-chronic liver failure (ACLF) is characterized by catastrophic loss of liver function in patients with advanced chronic liver disease, with 28-day mortality rates reaching up to 80%. Despite advances in intensive care, the high mortality primarily stems from the absence of a therapeutic modality that simultaneously addresses both the profound systemic inflammatory response and severe hepatic synthetic dysfunction.

**Methods:**

We designed an integrated extracorporeal circuit, termed the UTOpiA system, which combines granulocyte and monocyte apheresis (GMA) with human induced pluripotent stem cell-derived hepatocyte-like cell (iHLC) organoids engineered with HLA-A, HLA-B, and CIITA triple knockout. The efficacy of UTOpiA was tested after direct whole blood exposure through venous flow in rat models of ACLF and acute liver failure (ALF).

**Results:**

UTOpiA treatment significantly improved survival in both ACLF and ALF rat models, outperforming GMA or iHLC monotherapy and HepG2 cell-based devices. Improved survival was associated with reduced coma severity, improved liver biochemistry, and reduced hyperammonaemia, hyperbilirubinemia, and systemic inflammation. Transcriptomic and histological analyses revealed restoration of hepatic metabolic gene expression and hepatocyte regeneration. Mechanistically, iHLC-secreted α-fetoprotein suppressed hepatocyte cell cycle arrest via p21 downregulation and enhanced regeneration, while UTOpiA restored HNF4α activity and dampened pro-inflammatory cytokines, including IL-6 and TNF-α.

**Conclusions:**

The tandem UTOpiA circuit confers a significant survival benefit in preclinical rodent models of ACLF and ALF by providing anti-inflammatory, synthetic, and metabolic support. Elucidating the regenerative signals that promote recovery of the injured liver may further expand the potential of this whole blood extracorporeal system as a novel, off-the-shelf liver support therapy.

## Graphical abstract

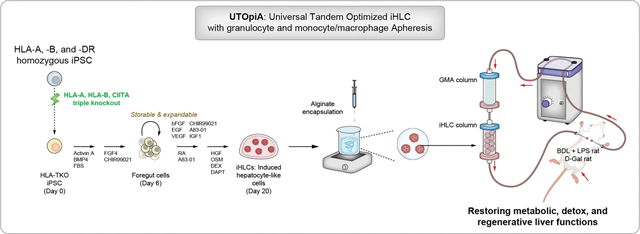

## Introduction

Acute-on-chronic liver failure (ACLF) is characterized by acute decompensation of cirrhosis and a high risk of short-term mortality in patients with multiorgan failure. ACLF is caused by an excessive systemic inflammatory response syndrome (SIRS) triggered by precipitants such as infection, heavy alcohol consumption, or gastrointestinal hemorrhage.^1,2^ This clinical entity is highly prevalent, affecting up to 35% of patients admitted with decompensated cirrhosis,^3^ and is associated with a poor prognosis, with 28-day mortality exceeding 50%.^4^ The main etiologies of the pre-existing chronic liver disease in ACLF include viral hepatitis, alcohol-related liver disease, and metabolic dysfunction-associated steatotic liver disease (MASLD), of which alcohol-related hepatitis and cirrhosis are the leading causes in Europe, according to the CANONIC study.^1^ Liver transplantation, although curative, is limited by the scarcity of donor organs, resulting in considerable mortality during the waitlist period.^5^ Despite advances in supportive intensive care, including management of underlying precipitants and complications such as sepsis, hepatic encephalopathy, and variceal hemorrhage, the mortality rate of ACLF remains unacceptably high and represents a major unmet medical need.

Extracorporeal systems that can mitigate the systemic inflammatory cascade would benefit patients with ACLF. A recent prospective, open-label, non-randomized pilot study demonstrated that granulocyte and monocyte/macrophage apheresis (GMA) improves 90- and 180-day survival of patients with corticosteroid-nonresponsive or -intolerant severe alcohol-related hepatitis, including patients with ACLF.^6^ While promising as a salvage anti-inflammatory therapy, the application to ACLF is limited by the lack of direct support of liver metabolic function.

Bioartificial liver (BAL) devices are extracorporeal circulation systems that aim to support hepatic functions of synthesis, detoxification, and metabolism for patients with liver failure. Combined with plasma separation, there have been historical attempts to use different types of hepatocytes in BAL devices, both in preclinical and clinical studies, including porcine hepatocytes,^7,8^ human hepatoma cell line-derived HepG2 cells,^9^ reprogrammed hepatocytes from human fibroblasts,^10,11^ virally-induced expandable primary hepatocytes,^12^ and hepatocyte-like cells differentiated from human induced pluripotent stem cells (iPSCs).^13^ The quest for sustainable sources of functional, safe, and massively expandable human hepatocytes still continues. Furthermore, although treatment with these BAL devices improved hepatic regeneration and survival in animal models of chemically induced and post-hepatectomy acute liver failure (ALF), the applicability of BAL devices for ACLF remains unclear. In addition to potential risks associated with allogenic or xenogeneic immune reactions, existing BAL systems do not directly alleviate acute systemic inflammation, a key pathogenic feature of ACLF.

We previously established iPSC-derived hepatocyte-like cell (iHLC) organoids from storable foregut progenitors^14,15^ that produce albumin and other plasma proteins at levels close to primary human hepatocytes (PHHs). These organoids are composed of apico-basolaterally polarized hepatocytes that enable directional uptake of bile acids and bilirubin, and exhibit key metabolic functions, including drug metabolism.^14–17^ Herein, we devised an extracorporeal whole blood circulation system comprised of a combination of iHLCs with GMA for the treatment of ACLF and ALF. We used genome-edited iPSC lines to generate liver organoids, wherein HLA (human leukocyte antigen)-A, HLA-B, and CIITA (class II major histocompatibility complex transactivator) were knocked out to enhance immunological compatibility to a broader population.^18^ Based on the dual composition of this system, which we have termed UTOpiA (Universal Tandem Optimized iHLC with granulocyte and monocyte/macrophage Apheresis), we hypothesized that its use in rat models of ACLF and ALF would lead to improvements in liver function, optimally control systemic inflammation, and promote a hepatic regenerative response, ultimately improving survival.^19^

## Materials and methods

### Human iPSC lines and cell culture

The Ff-XT28s05_cont human iPSC line (HLA homozygous iPSC line from a male donor DRXT with the third most frequent haplotype in Japan), and hypoimmunogenic iPSC lines Ff-XT28s05-Abo_To #14–4 from a donor DRXT and Ff-I01s04-AbII KO-54 from a male donor QHJI (the first most frequent haplotype in Japan), in which HLA-A, HLA-B, and CIITA genes were depleted by CRISPR-Cas9 genome editing^18,20^, were obtained from the CiRA Foundation and used for this study with approval from the Ethics Committee at Faculty of Medicine of Institute of Science Tokyo (M2021–162) and The University of Osaka Hospital Ethical Review Board (23160). All human iPSC lines were cultured with StemFit AK02N (Ajinomoto Co, Japan) and iMatrix-511 (Nippi, Japan) at 37 °C in 5% CO_2_ with 95% air. Cryopreserved PHHs, which were validated for their spheroid formation capacity, were purchased from Lonza. HepG2 C3A cells were purchased from ATCC and were maintained in DMEM (low glucose; nacalai tesque) supplemented with 10% FBS.

### Generation of iHLCs

All described cell cultures were performed at 37 °C in 5% CO_2_ with 95% air and in 100% moisture. Human iPSCs were differentiated into foregut (FG) using previously described methods.^14^ In brief, human iPSCs were detached by Accutase (Thermo Fisher Scientific) and were seeded on iMatrix-511-coated tissue culture plates with 100,000 cells/cm^2^. Medium was changed to RPMI 1640 medium (nacalai tesque) supplemented with 1× non-essential amino acids (Thermo Fisher Scientific) that contain 100 ng/ml activin A (R&D Systems), 50 ng/ml bone morphogenetic protein 4 (BMP4; R&D Systems), and 0.25% B27 (gibco) at day 1, 100 ng/ml activin A and 0.2% fetal bovine serum (FBS; Thermo Fisher Scientific Inc) at day 2, and 100 ng/ml activin A and 2% FBS at day 3. On days 4 to 6, cells were cultured in Advanced DMEM/F12 (Thermo Fisher Scientific) with 1× B27 (Life Technologies) and 1× N2 (Gibco) containing 500 ng/ml fibroblast growth factor 4 (FGF4; R&D Systems) and 3 μM CHIR99021 (Stemgent) to induce the FG. The medium was replaced daily. The obtained FG cells were detached by Accutase and then frozen in the CELL-BANKER 1 freezing medium (Nippon Zenyaku Kogyo Co, Ltd), and kept at −80 °C or in liquid nitrogen for long-term storage.

To generate iHLCs from the FG, FG cells were quickly thawed and then centrifuged at 300 ×g for 5 min. They were resuspended in the Matrigel (Corning) on ice with approximately 100,000 viable cells being embedded in 50 μl of the Matrigel drop, and subsequently grown in Advanced DMEM/F12 with 2% B27, 1% N2, 1× HEPES, 1× GlutaMAX, 1% Pen/Strep, 3 μM CHIR99021, 20 ng/ml epidermal growth factor (EGF), 1 μM A83–01, 10 ng/ml vascular endothelial growth factor (VEGF), 5 ng/ml basic fibroblast growth factor (bFGF), 25 ng/ml insulin-like growth factor 1 (IGF1), and 50 μg/ml ascorbic acid. Following culture with Y-27632 for 3 days and an additional 2–3 days without Y-27632 to form the spheroids, the medium was replaced with Advanced DMEM/F12 with 2% B27, 1% N2, 1× HEPES, 1× GlutaMAX, 1% Pen/Strep, 2 μM retinoic acid 1 μM A83–01 for 2 days and subsequently without A83–01 for further 2 days with medium changed every other day. The generated organoids were isolated from Matrigel and then cultured in the suspension culture in HCM Hepatocyte Culture Medium BulletKit (Lonza; human EGF singlequot was removed) supplemented with 100 nM dexamethasone, 20 ng/ml Oncostatin M, 10 ng/ml hepatocyte growth factor (HGF), 10 μM DAPT, and 10% Matrigel at the ultra-low attachment 6-well plate (Thermo Fisher Scientific) for 10–14 days, with medium changed every 3 days.

### PHHs, PHH spheroids and HepG2 C3A spheroids

Cryopreserved PHHs were thawed and resuspended in the spheroid formation medium, which consists of HCM Hepatocyte Culture Medium BulletKit (Lonza; human EGF singlequot was included) with 20% FBS and 25 mM HEPES. For 3D spheroid culture, the cells were plated into 96-well ultra-low attachment plates (Corning) at a density of 1,500 cells/well. Spheroid formation was confirmed 2–3 days after seeding. The PHH spheroids were transferred into the 6-well ultra-low attachment plate (ThermoFisher Scientific) followed by medium change every 2–3 days. The spheroids were used until day 10 for the indicated experiments.

HepG2 spheroids were generated by using EZSPHERE 6-well microplate 900 (AGC), according to the manufacturer’s protocol. The cells were seeded at a density of 5.4×10^5^ cells/well, resulting in the formation of approximately 2,700 spheroids per well. Spheroids formed 2 days after seeding were used for the indicated experiments.

### Flow cytometry analysis of iHLCs

To analyze the surface expression of HLA, iHLCs were dislodged from Matrigel and transferred into low-attachment 6-well plates (Thermo Fisher Scientific) followed by treatment with or without 25 ng/ml interferon-γ (IFN-γ; Proteintech) for 24 h. The organoids were then collected and treated on ice with Cell Recovery Solution (Corning) for 10 min to dissolve the remaining Matrigel. They were collected in the wash buffer (DMEM/F12 supplemented with 0.1% BSA fraction V) and further digested into single cells using TrypLE Express Enzyme (Gibco) for 15–20 min at 37 °C water bath. The dissociated cells were collected in the wash buffer and centrifuged at 300 ×g for 10 min. Each cell pellet was resuspended in Cell Staining Buffer (BioLegend) with Ghost Dye Violet 450 (TONBO). For staining, the suspension of cells was incubated with 100 μl of diluted antibodies for 30 min on ice. After washing, samples were analyzed on a BD LSR Fortessa (BD Biosciences), and the data were processed using FlowJo software (Ver. 10.10.0). Anti-HLA-A24 mAb-Alexa Fluor 647 (MBL) was used for HLA-A staining. Anti-HLA-B7 antibody (Bio-Rad) followed by secondary staining with Goat Alexa Fluor 647 anti-mouse IgG antibody (Invitrogen) was used for HLA-B staining.

### Measurement of albumin secretion and urea production

To determine albumin secretion and urea production in organoids, organoid culture supernatants were collected and stored at −80 °C until use. The supernatants were assayed with Human Albumin ELISA Quantitation Set (Bethyl Laboratories) and QuantiChrom Urea Assay Kit (BioAssay Systems).

### Measurement of glucose production in iHLCs

Glucose production in iHLCs was measured using the Glucose-Glo-Assay (Promega) according to the manufacturer’s protocol. The organoids were rinsed three times with cold PBS, resuspended in DMEM without glucose supplemented with 100 μM sodium pyruvate (Gibco), and seeded into low-attachment 6-well plates (Thermo Fisher Scientific). After 24 h, the culture supernatant was collected and used for glucose measurement. The luminescent signal was measured using a FLUOstar OPTIMA-6 microplate reader (BMG Labtech). To estimate the number of the organoids assayed and normalize the produced glucose level, the bright-field image of each well was captured using a Cell3Imager duos2 (SCREEN), and the organoid area at a single organoid level was automatically segmented using a model trained using the implemented deep learning.

### Measurement of bilirubin uptake in iHLCs

Hyperbilirubinemic plasma was obtained from bile duct ligated rats. Alginate-encapsulated iHLCs and PHH spheroids were loaded into the column and plasma was circulated *ex vivo* by a peristaltic pump for 2 h. The exposure of alginate beads alone to the plasma was tested as a control to obtain the net amount of bilirubin uptake by iHLCs and PHHs.

### Histology, immunohistochemistry, and immunofluorescence

Liver samples were fixed overnight in 4% PFA and embedded in paraffin blocks. After deparaffinisation, H&E and immunohistochemical staining were performed. The dilution magnification of the antibodies is shown in Table S1. Samples stained with H&E and peroxidase-based immunohistochemistry were imaged with a BZ-X800 microscope (KEYENCE). The immunofluorescent images were obtained with a SP8 confocal microscope (Leica) with HyD detectors, or with a THUNDER 3D Live Cell Imaging system (Leica) for tile scanning of wider fields. For whole-mount staining of iHLCs, encapsulated iHLCs were fixed overnight at 4 °C in 4% paraformaldehyde. iHLCs were washed with PBS, 1 mM EDTA was added, and pipetted and centrifuged at 300 × g for 5 min to remove supernatant. After the primary antibody, the iHLCs were washed three times with wash buffer (0.1% Triton-X 100/PBS). After the secondary antibody reaction, iHLCs were washed, visualized, and scanned with a SP8 confocal microscope with HyD detectors, or with a FV3000 confocal microscope (EVIDENT). The dilution magnification of the antibodies is shown in Table S1.

### Rats

Male Sprague-Dawley rats (body weight 300–350 g; age 8–11 weeks) were obtained from the Sankyo laboratory. All rats were maintained in our specific pathogen–free animal facility, and all rat experiments were approved by the Institutional Animal Care Committee of Institute of Science Tokyo and The University of Osaka. Rats that had bleeding of more than 3 ml before or after extracorporeal circulation were excluded from the study. All animals that reached a moribund state, defined as Grade 4 hepatic encephalopathy, weight loss of more than 25% in 1 week, and/or cachexia, were euthanized in accordance with ethical policy for laboratory animals.

### Common bile duct ligation (BDL) and induction of endotoxemia by LPS administration

Secondary biliary cirrhosis was induced in male Sprague-Dawley rats (300–350 g of body weight) by ligating the common bile duct for 21 days. While each animal was under anesthesia, the common bile duct was occluded by double ligation with 5–0 silk thread. The bile duct was then cut again between the two ligature threads. Two days after the operation, a change in urinary bilirubin to dark brown was considered indicative of successful ligation. Acute endotoxemia induced by intraperitoneal administration of lipopolysaccharide (LPS) (from *Escherichia coli* O111:B4, Sigma-Aldrich) was used to induce SIRS, causing an acute deterioration of cirrhosis and mimicking human ACLF. An LPS dose of 0.25 mg/kg was selected based on the literature^19,21^ and administered 1 h prior to initiation of the extracorporeal circulation.

### BAL system

iHLC alginate capsules (6–10 capsules) containing approximately 3 million cells and 3 mg per g body weight of 300 μm cellulose acetate beads were separately packed into two 1 ml columns. Each column was placed in series and a circuit was assembled. The circuit was connected to a peristaltic pump and the tubing and column were filled with heparinized saline. 26G intravenous catheters were placed in the left and right external jugular veins of rats under continuous anesthesia by isoflurane and connected to the circuit.

### BAL treatment in rats

In the Empty and UTOpiA groups, extracorporeal circulation was initiated 1 h after LPS administration; sedation was provided with 2% isoflurane during BAL treatment. The circuit was primed with heparinized saline (10 units/ml heparin), followed by a bolus dose of 200 units of heparin via catheter at the start. Rats were connected to the extracorporeal device via left and right jugular vein catheters. Peristaltic pumps were set at a flow rate in 1 ml/min according to a previous study of septic rats treated with GMA.^22^ The rats underwent 2 h of extracorporeal circulation. After BAL treatment, rats were awakened, caged, and followed for 3 days. Blood samples were collected before and after BAL treatment. Plasma toxin (bilirubin and ammonia) and inflammatory cytokine levels were measured. Every day afterward, blood was sampled until the animals were sacrificed at the endpoint. Coma severity was assessed at 3 and 24 h after treatment according to standardized coma scaling for rats.^6^ The following reflexes, physiological responses, and behavioral domains were assessed: motor function (reflexivity, purposefulness), brainstem reflexes (pupillary, corneal, and fin reflexes), breathing, righting reflexes, auditory responses, and whisker movements.

### RNA-seq analysis

Total RNA was extracted from snap-frozen rat liver tissues using a two-step protocol. Briefly, the frozen tissues were homogenized in the TRI reagent (Molecular Research Center) followed by addition of chloroform and mixing for phase separation. After centrifugation, the aqueous phase was isolated, which was further re-extracted with FastGene RNA Premium Kit (NIPPON Genetics) including DNase I treatment. The RNA-sequencing (RNA-seq) libraries were prepared using MGIEasy RNA Directional Library Prep Set V2.0 (MGI Tech) with polyA selection. Subsequently, the libraries were sequenced using a paired-end 150-bp protocol on a DNBSEQ-G400 (MGI Tech). The quality of the raw paired-end sequence reads was assessed with FastQC (v0.10.1). Low quality (the 5’ and 3’ end bases that contains N’s or of quality values below 20, and reads that are less than 75 bp long after trimming) and adapter sequences were filtered by Cutadapt software (version 1.9.1). The trimmed reads were aligned to the reference genome (GRCm38 (mm10)) with HISAT2 (v2.0.1) with default parameters. The bam files were used to estimate the abundance of uniquely mapped reads with featureCounts (version 2.6.0). The raw read counts were normalized and processed for differential gene expression analysis using DESeq2 (v1.42.0) in R software (v4.2.3). Statistical significance of genes was determined by an adjusted *p* <0.05 and |fold change| >2. Gene ontology analysis was performed on the differentially expressed genes using enrichGo in the clusterProfiler R package (v4.10.0). Gene set-enrichment analysis for the TPM normalized gene counts was performed using the GSEA software (v4.1.0), the Enrichr, and the fgsea package. Transcription factor activity was inferred from bulk RNA-seq data using the decoupleR package.

### Plasma biochemistry

Plasma samples collected from randomly selected rats were subjected to the Rat Cytokine Array (RayBiotech) containing antibodies to 48 cytokines according to the manufacturer’s instructions. Collected rat plasma was assayed using the HGF ELISA kit (R&D, MHG00), Human Albumin ELISA Quantitation Set (Bethyl Laboratories), and α-fetoprotein (AFP) ELISA kit (RayBiotech, ELH-AFP-1), according to the manufacturers’ protocols. Biochemical tests such as aspartate aminotransferase (AST), alanine aminotransferase (ALT), ammonia, and total bilirubin were measured using DRI-CHEM NX500V (Fujifilm, Japan).

### Statistical analysis

Comparisons between two groups were performed using unpaired two-tailed Student’s *t* test, whereas comparisons among more than two groups were analyzed by analysis of variance (ANOVA) and Tukey post hoc tests. Mantel-Cox log-rank test was used for survival analysis. *p* <0.05 was considered statistically significant. Statistical analyses were performed using GraphPad Prism 10 (GraphPad Software).

## Results

### Generation of iHLCs from HLA-A, B, and CIITA triple knockout iPSCs

Recent reports have demonstrated the potential of hypoimmune, allogeneic iPSC-based products for off-the-shelf immune cell therapy.^23–26^ Given that ACLF progresses rapidly, we nominated HLA-A, HLA-B, and CIITA triple knockout (TKO) iPSC lines for further development in UTOpiA because well-characterized, clinical-grade lines are readily available.^20^

**Fig. 1.**
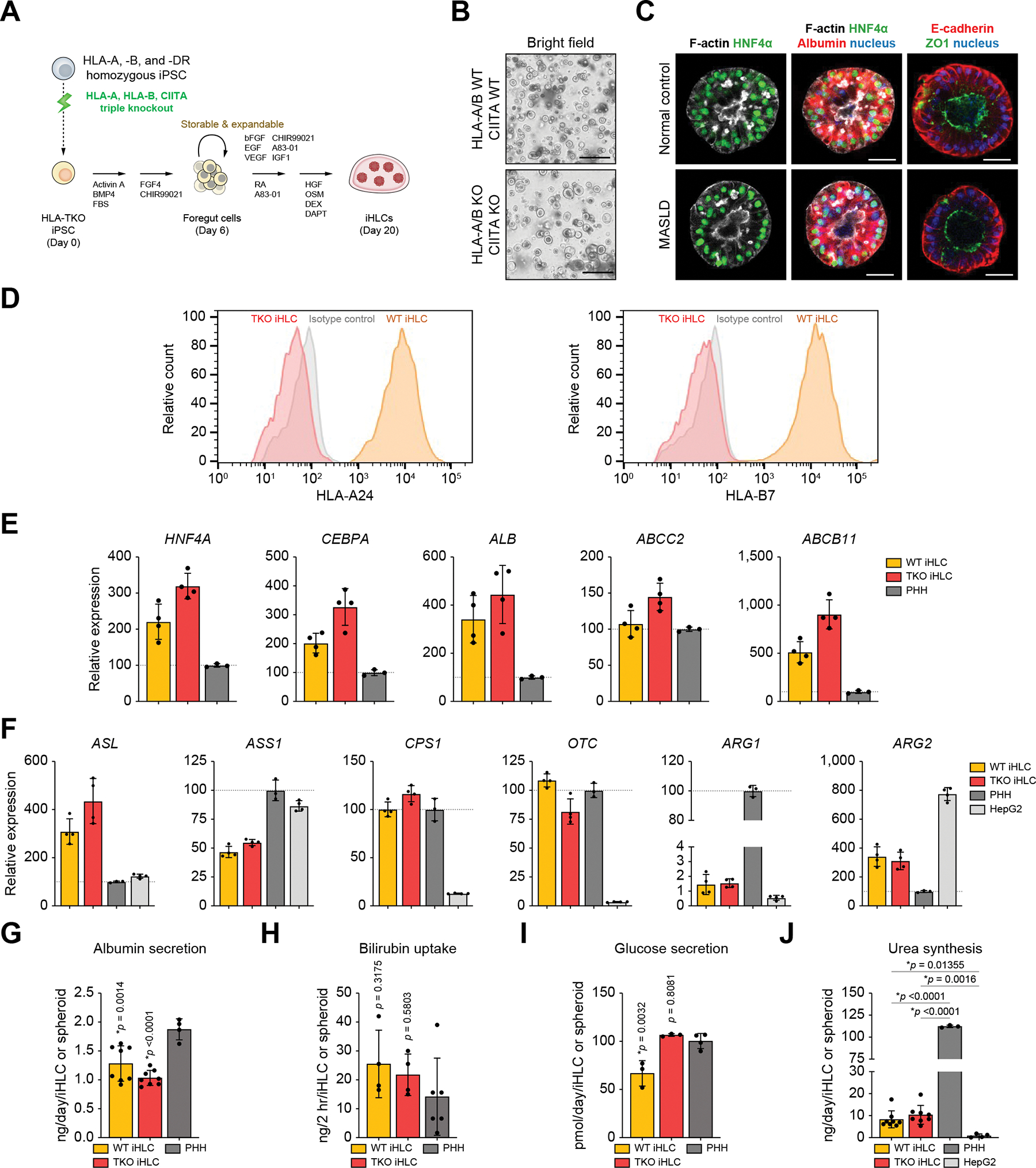
Generation of iHLCs from HLA-A, B, CIITA triple knockout iPSCs. (A) Schematic diagram of generation of iHLCs from HLA-A, B, CIITA TKO iPSCs. (B) Representative bright-field images of TKO iPSC-derived iHLCs as well as the parental WT cells. Scale bar, 400 μm. (C) Whole-mount immunostaining of iHLCs to visualize F-actin, HNF4α and albumin (left, middle), and E-cadherin and ZO-1 (right). Nuclei were counterstained. Scale bar, 50 μm. (D) Flow cytometry of WT and TKO iHLCs treated with IFN-γ showing the cell surface levels of HLA-A24 and HLA-B7. (E) RT-qPCR analysis of gene expression related to hepatocyte markers in WT and TKO iHLCs. The expression levels of PHH spheroids were also compared. Data are shown as mean ± SD normalized to PHHs. (F) RT-qPCR analysis of gene expression related to urea cycle in WT and TKO iHLCs. The expression levels of PHH and HepG2 spheroids were also compared. Data are shown as mean ± SD normalized to PHHs. (G,H,I) Quantitative analysis of albumin secretion, bilirubin uptake, and glucose secretion of WT and TKO iHLCs based on the measurement of culture supernatants *in vitro*. PHH spheroids were added as a control. Data are shown as mean ± SD with *p* values *vs.* PHH (Tukey’s *post hoc* test). (J) Measurement of urea synthesis in WT and TKO iHLCs as well as PHH and HepG2 spheroids. Data are shown as mean ± SD with *p* values of the indicated comparisons (Tukey’s *post hoc* test). iHLC, induced hepatocyte-like cell; IFN-γ, interferon-gamma; PHH, primary human hepatocyte; RT-qPCR, reverse transcription quantitative PCR; TKO, triple knockout; WT, wild-type.

We previously developed a differentiation protocol to derive FG cells from iPSCs, which can be cryopreserved and expanded.^14,15^ With minor modifications, we produced hepatocyte-like cell aggregates from HLA-A, HLA-B, and CIITA TKO iPSC lines, hereafter described as iHLCs (Fig. 1A). The differentiated FG cells and iHLCs derived from the TKO iPSC line were morphologically similar to those from the parental iPSC line (Fig. 1B and S1A). The iHLCs from both lines expressed hepatocyte-specific markers ALB and HNF4α with the polarized epithelium positive for F-actin and ZO-1 in the intraluminal region (Fig. 1C). The loss of surface presentation of HLA in the TKO iHLCs was confirmed by boosting HLA surface expression with interferon-γ (IFN-γ) for 24 h. WT iHLCs exhibited increased surface expression of both HLA-A24 and HLA-B7, which were depleted in TKO cells (Fig. 1D).

Gene expression in HLA-A, HLA-B, and CIITA TKO iHLCs and wild-type iHLCs was profiled by quantitative PCR relative to PHH spheroids (Fig. 1E and S1B). First, we evaluated the state of PHH spheroids by comparison with conventional 2D monolayer culture. PHH spheroids exhibited ALB expression similar to 2D cultures and secreted 1.25 ± 0.121 μg of albumin per day per 1 × 10^6^ cells, about 79% of the 2D level (Fig. S1C and D). Spheroid culture increased the expression of ARG1, a rate-limiting enzyme in the urea cycle, and enhanced urea synthesis up to 5.6-fold (Fig. S1C and E). Hepatic lineage marker genes were highly expressed in both WT and TKO iHLCs, such as *HNF4A* (221% in WT and 320% in TKO *vs.* PHHs), *CEBPA* (202% in WT and 327% in TKO *vs*. PHHs), and *ALB* (342% in WT and 445% in TKO *vs.* PHHs). Expression of hepatocyte transporters *ABCC2* (108% in WT and 145% in TKO *vs.* PHHs) and *ABCB11* (512% in WT and 905% in TKO *vs*. PHHs), which encode multidrug resistance-associated protein 2 (MRP2) and bile salt export pump (BSEP), respectively, were comparable to or even greater in iHLCs than in PHHs. We also analyzed the expression of genes involved in the urea cycle, of which metabolic capacities are generally lost in HepG2 cells and compromised in most HLCs.^27^ Here, expression of most of these genes, including *CPS1, OTC, ASL,* and *ARG2* was comparable to or even higher in both WT and TKO iHLCs compared with PHHs, except for *ARG1*, which was almost absent in HepG2 cells (Fig. 1F).

Albumin production was similar between the HLA-A, B, CIITA WT and TKO iHLCs (1.28 ± 0.31 ng/day/iHLC and 1.03 ± 0.13 ng/day/iHLC, *p* = 0.1020), compared with 1.88 ± 0.18 ng/day/PHH spheroid (Fig. 1G). Both WT and TKO iHLCs were able to excrete a fluorescent bile acid analogue into their lumen (Fig. S1F). By exposing the cells *ex vivo* to hyperbilirubinemic plasma from BDL rats, we measured the bilirubin uptake of iHLCs. Bilirubin uptake was comparable in both WT and TKO iHLCs and in PHH spheroids (Fig. 1H). When compared with WT iHLCs, TKO iHLCs secreted higher levels of glucose into an extracellular milieu, similar to that of PHHs (Fig. 1I). Consistent with the limited expression of *ARG1*, which mediates the final reaction in the urea cycle, iHLCs produced less urea than PHHs, yet significantly more than HepG2 cells (Fig. 1J). These results suggest that the HLA-A, HLA-B, and CIITA genome-edited iPSCs successfully differentiate into iHLCs.

### Development of the UTOpiA system

**Fig. 2.**
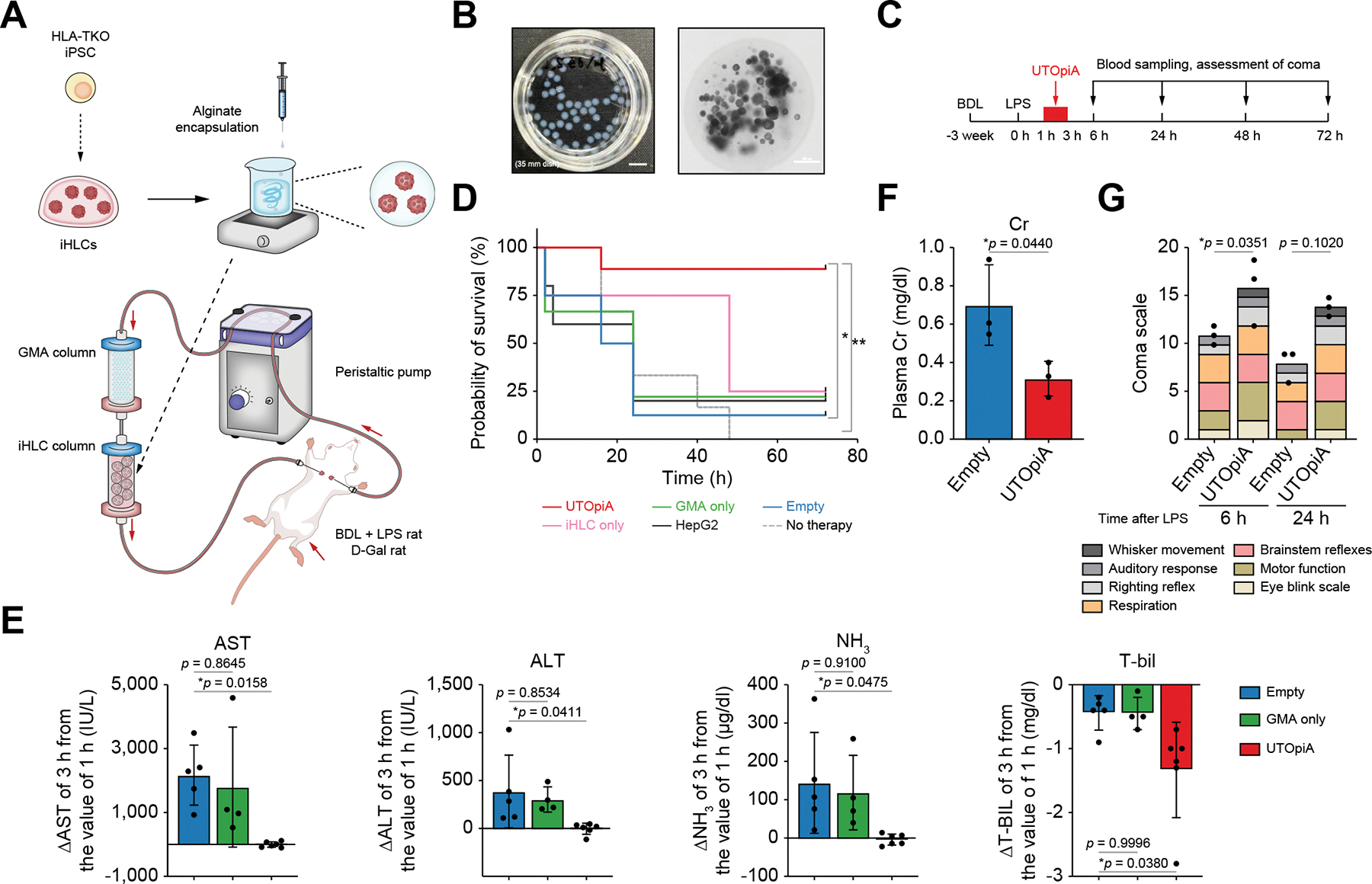
Extracorporeal whole blood circulation system with alginate-encapsulated iHLCs and GMA improves survival of ACLF rats. (A) Schematic diagram depicting the configuration of the UTOpiA system, where the GMA and alginate-encapsulated HLA-TKO iHLCs columns were connected in tandem to the ACLF model rat. (B) Representative images of alginate-encapsulated iHLCs. Scale bar, 5 mm (left), 500 μm (right). (C) Schematic diagram of the treatment protocol. One hour after intraperitoneal LPS injection into rats with BDL, UTOpiA was performed for 2 h. (D) Kaplan-Meier survival curve of BDL+LPS rats treated with no therapy (n = 6), empty column (n = 8), GMA column only (n = 9), iHLC column only (n = 4), and tandemly connected GMA-HepG2 columns (represented as HepG2 in the graph; n = 5), GMA-iHLC columns (n = 9). Statistical significance of the two groups was tested by log-rank (Mantel-Cox) test followed by Bonferroni correction. *p* values that met the significance level after the correction (0.0033) were indicated with asterisks (*, **). (E) Relative changes in plasma levels of AST, ALT, ammonia and T-bil at the end of the BAL treatment compared with levels at 1 h. The values of differences before and after the treatment are shown as average ± SD (Tukey’s *post hoc* test). (F) Plasma creatinine levels at the 3 h after the BAL treatment. Average ± SD is shown (Student’s *t* test). (G) Coma severity was assessed at 6 and 24 h after LPS induction according to the coma scoring for rats including eye blink, motor function, brainstem reflexes, respiration, righting reflex, auditory response, and whisker movement (Student’s *t* test). ACLF, acute-on-chronic liver failure; ALT, alanine aminotransferase; AST, aspartate aminotransferase; BAL, bioartificial liver; BDL, bile duct ligation; GMA, granulocyte and monocyte apheresis; iHLC, induced hepatocyte-like cell; LPS, lipopolysaccharide; TKO, triple knockout; T-bil, total bilirubin.

We next sought to devise iHLC housing and assembled the BAL system for whole blood exposure in rats (Fig. 2A). Most current BAL systems adopt a plasma component separator to isolate the cellular reservoir from host blood cells, and expose the separated plasma to hepatocytes.^28^ These devices require a complex design, such as an oxygenator and air-liquid interface to support hepatocyte viability and metabolic function.^9,12^ To avoid these system complexities and ensure experimental feasibility in preclinical rodent experiments,^28^ we developed an extracorporeal whole blood circulation system including iHLCs in combination with a GMA column. To allow for whole blood exposure, we first encapsulated iHLCs with alginate, selected due to its high biocompatibility and physical stability.^29^ This approach provides a semipermeable barrier for substance exchange, as well as physical protection from host blood immune cell-mediated damage (Fig. 2B). The encapsulated iHLCs maintained their viability, and albumin secretion was comparable to the cells before encapsulation (Fig. S2A). We next combined the iHLC column with a GMA column to remove circulating neutrophils and monocytes/macrophages that drive systemic inflammation associated with ACLF (Fig. 2A). We found that a tandem connection with the GMA column upstream, but not downstream, of the iHLC housing column offered post-exposure cytoprotection in alginate-encapsulated iHLCs after extracorporeal whole blood circulation in rats (Fig. S2B and C). By canulating the jugular veins both for transfer and return of whole rat blood circulated via a peristaltic pump, we established a system consisting of a GMA column and hypoimmunogenic, iPSC-derived iHLC column in tandem, termed Universal Tandem Optimized iHLC with granulocyte and monocyte/macrophage Apheresis, or UTOpiA.

### UTOpiA rescues rats from BDL-induced decompensated ACLF

To characterize the contribution of the two components of the UTOpiA system in the tandem circuit and to establish clinical proof of concept, we explored its survival benefit in a sepsis-related, preclinical model of ACLF in rats with BDL-induced biliary cirrhosis.^21^ BDL rats were divided into six treatment groups following the intraperitoneal injection of LPS to induce endotoxemia: no therapy; treatment with the system containing neither cells nor GMA (empty group); treatment with the system containing either iHLCs only or GMA only (iHLC only group; GMA only group); treatment with the system containing HepG2 cells and GMA (HepG2 group); and treatment with the UTOpiA system (UTOpiA group). One hour after LPS administration, each rat underwent 2 h of extracorporeal whole blood circulation (as per the groups defined above) and was monitored for up to 3 days thereafter (Fig. 2C). On the initiation of extracorporeal circulation, vital signs were stable and circulatory access was established via the internal jugular veins. Consistent with a previous study,^21^ very high mortality was observed in the no therapy and empty groups within 24 h after LPS administration, with 0% and 12.5% survival, respectively, at the study endpoint (Fig. 2D). A surviving rat in the empty group demonstrated behavioral deficits presumably due to prolonged hepatic dysfunction at 24 h. Among the groups receiving active treatment, the highest survival was observed in the UTOpiA group (88.9%), which was significantly higher than in the iHLC only group (25.0%) and the GMA only group (22.2%). Only 20.0% of rats in the HepG2 group (treated with HepG2 cells and the GMA column) survived, supporting the enhanced efficacy of the iHLCs over HepG2 cells.

Biochemical measurements of rat plasma before and after the extracorporeal circulation demonstrated that UTOpiA treatment mitigated increases in plasma AST, ALT, and ammonia induced by BDL and LPS administration (Fig. 2E); this downward trend with UTOpiA treatment persisted for up to 72 h following LPS administration (Fig. S3A). UTOpiA treatment significantly reduced total bilirubin concentrations compared with empty treatment. No improvements in plasma AST, ALT, or ammonia concentrations were observed in the HepG2 group nor the GMA only group (Fig. S3B). Endotoxemia in cirrhotic rats induces mild renal injury.^21^ Indeed, UTOpiA treatment reduced plasma creatinine levels compared to empty treatment (Fig. 2F). Rats in the empty group developed hepatic encephalopathy as evidenced by the onset of coma at 3 h after LPS administration. By employing the Tübingen-Boston rat coma scale,^30^ we found that UTOpiA treatment improved the behavioral phenotypes with significant improvement in clinical signs and symptoms of coma 6 h after LPS administration; a similar trend was observed after 24 h (Fig. 2G). These data indicate that the iHLCs and GMA in UTOpiA offer a survival benefit in this rat model of ACLF by ameliorating both the acute hepatic insult and subsequent multiorgan failure.

We next evaluated the extent of iHLC damage after completion of extracorporeal circulation in ACLF rats. At 2 h post completion, we found that the tandem connection with the GMA column significantly improved the survival of iHLCs, accompanied by reduced apoptosis, as assessed by calcein-AM/EthD-1 and cleaved caspase-3 staining (Fig. S2D and E). A protective effect of the GMA column on the iHLCs was supported by reduced translocation of cell-free human mitochondrial DNA into rat plasma during the extracorporeal circulation (Fig. S2F). These data support the cytoprotective role of the upstream GMA column for the downstream iHLCs, effectively enabling direct whole blood exposure without compromising the viability of iHLCs.

### UTOpiA treatment ameliorates hepatocyte injury in ACLF rats

**Fig. 3.**
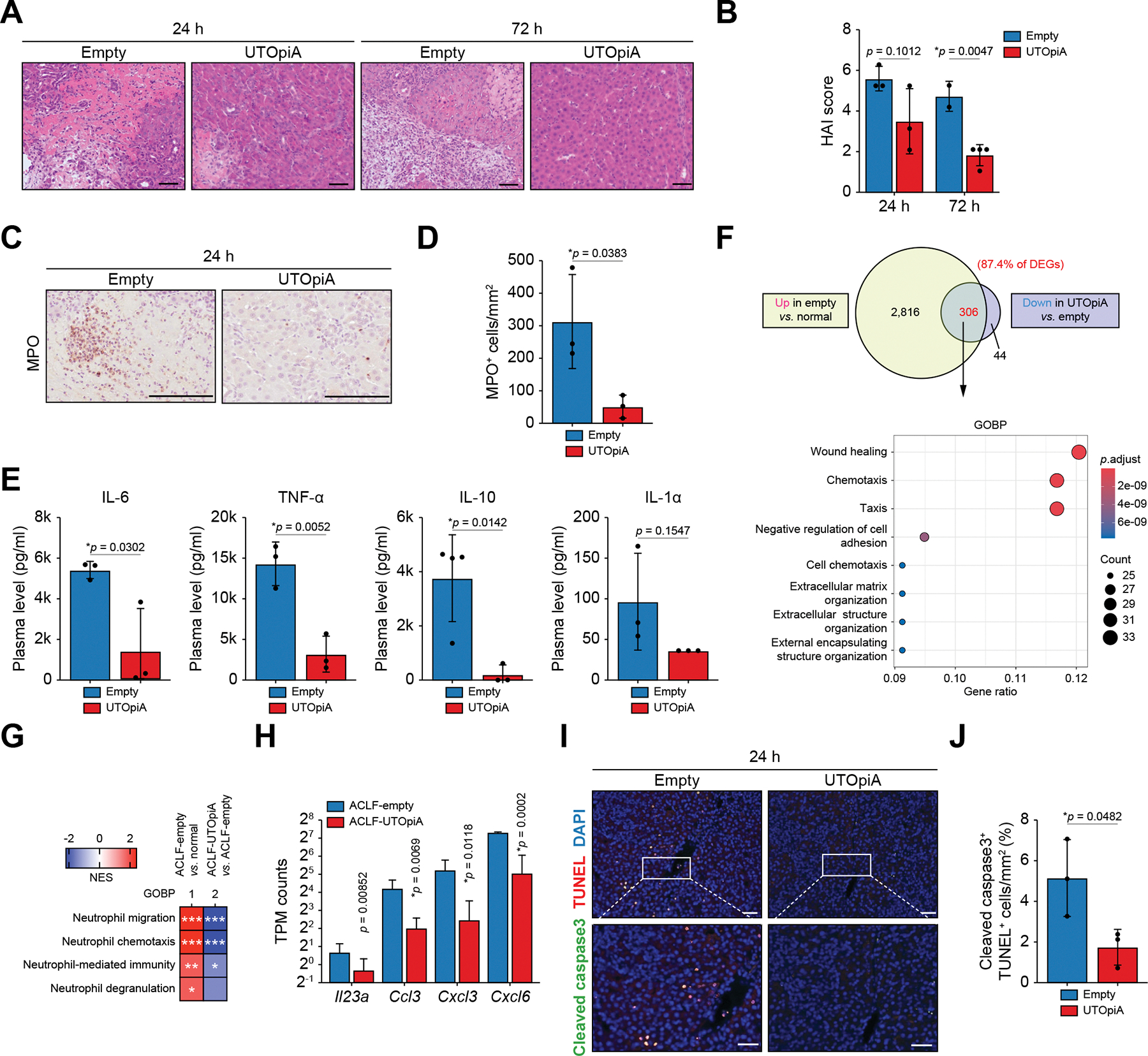
UTOpiA treatment ameliorates hepatocyte injury in ACLF rats. (A) Representative images of H&E staining of liver tissues in the BDL+LPS rats in empty column- and UTOpiA-treated groups (24 and 72 h after LPS induction). Scale bar, 50 μm. All the images shown were derived from different animals. (B) HAI score of liver histology of BDL+LPS rats in empty column- and UTOpiA-treated groups. Average ± SD is shown (Student’s *t* test). (C,D) Improvement of neutrophil infiltration into liver tissues 24 h after the UTOpiA treatment indicated by MPO immunohistology (C). Scale bar, 100 μm. Quantification of the number of MPO-positive cells (D). Data are shown as mean ± SD (Student’s *t* test). (E) Plasma levels of IL-6, TNF-α, IL-10, IL-1a at the end of the treatment in empty column- and UTOpiA-treated groups. Average ± SD is shown (Student’s *t* test). (F) Venn diagrams showing the number of upregulated genes in liver tissues of BDL+LPS rats with empty treatment *vs.* normal rat, and of downregulated genes in those of BDL+LPS rats with UTOpiA treatment *vs.* with empty treatment. The overlapping regions indicate the number of DEGs reverted by UTOpiA treatment. GO analysis of biological processes for the reverted genes is also shown. (F) Venn diagram and GO analysis showing downregulated GOBP terms in UTOpiA *vs.* Empty groups. (G) Gene set-enrichment analysis showing the differential GOBP terms related to neutrophils between the ACLF empty column-treated group and the normal group, as well as between the empty column- and UTOpiA-treated groups. *p <0.05; **p <0.01; ***p <0.001. (H) The gene expression of neutrophil-attracting cytokines in empty column- and UTOpiA-treated groups based on RNA-seq data (Sidak’s *post hoc* test). (I,J) Representative images of TUNEL and cleaved caspase-3 immunostaining of liver tissues of BDL+LPS rats in empty column- and UTOpiA-treated groups (24 h after LPS induction) (I). Scale bar, 50 μm. Quantification of the number of cells positive for cleaved caspase-3 and TUNEL (J). Average ± SD is shown (Student’s *t* test). ACLF, acute-on-chronic liver failure; BDL, bile duct ligation; DEGs, differentially expressed genes; GO, gene ontology; HAI, histological activity index; LPS, lipopolysaccharide; MPO, myeloperoxidase; RNA-seq, RNA-sequencing.

To analyze whether systemic inflammation-induced liver injury was restored after UTOpiA treatment, surviving rats in either the empty or UTOpiA groups were randomly selected and sacrificed 24 and 72 h after the administration of LPS. Histological analyses revealed the emergence of hepatocellular necrotic areas associated with intrahepatic inflammation and hemorrhage, which was ameliorated by UTOpiA treatment (Fig. 3A). Improvement of the histological activity index^31^ with UTOpiA was observed as early as 24 h after LPS administration, with continued and statistically significant improvement at 72 h (Fig. 3B). In the ACLF rat model used in this experiment, myeloperoxidase (MPO)-positive neutrophils infiltrated the liver 3 h after LPS administration and remained for up to 24 h (Fig. S3C). Neither the GMA only or HepG2 groups demonstrated improvements in hepatocyte necrosis or inflammation (Fig. S3D). The number of MPO-positive cells in the necrotic zone was significantly reduced in the UTOpiA group compared with the empty group (Fig. 3C,D). UTOpiA treatment also reduced plasma levels of a neutrophil-produced cytokine, IL-1α,^32^ and a neutrophil recruitment factor, IL-10.^33^ Moreover, the proinflammatory cytokines IL-6 and TNF-α were downregulated by UTOpiA treatment, along with a concomitant decrease in the inflammatory mediators VEGF and β-NGF (Fig. 3E and S4A).

To gain further insight into the molecular basis underlying the improvement in liver inflammation and injury with UTOpiA treatment, we performed bulk tissue RNA-seq analysis of normal and ACLF livers in the empty and UTOpiA treatment groups at 24 h after LPS induction. Compared with ACLF livers of the empty group, there were 229 and 350 differentially expressed genes (DEGs) upregulated and downregulated in livers of the UTOpiA group, respectively (Fig. S4B). The majority (87.4%) of the downregulated DEGs (304 genes) were upregulated genes associated with ACLF compared to normal livers (Fig. 3F). These genes were related to wound healing and chemotaxis as revealed by gene ontology (Fig. 3F and S4C). Significant reductions in hepatic mRNA levels of *Infg*, *Tnf*, *Il6*, and *Ilb* were observed in the livers of UTOpiA-treated rats (Fig. S4D). Complementing our histological observations (Fig. 3C, D), gene set-enrichment analysis revealed significant upregulation of neutrophil activation pathways – including migration, chemotaxis, and degranulation – in the livers of ACLF rats in the empty group, compared to downregulation in the UTOpiA group (Fig. 3G). In the UTOpiA group, genes that activate neutrophil chemotaxis, including *Il23a*, *Ccl3*, *Cxcl2*, and *Cxcl6*, were suppressed compared to the empty group (Fig. 3H). Immunofluorescence staining of liver tissues harvested at 24 h post treatment in the empty or UTOpiA groups showed that cells positive for both cleaved caspase-3 and TUNEL were decreased by UTOpiA treatment (Fig. 3I, J). These findings suggest that one mechanism by which UTOpiA treatment exerts its effects is by inhibiting neutrophil recruitment, thereby protecting hepatocytes.

### UTOpiA system rescues rats from D-galactosamine-induced ALF

To evaluate the efficacy of UTOpiA in another condition characterized by acute hepatic deterioration, we applied UTOpiA treatment in rats with D-galactosamine (D-Gal)-induced ALF. Briefly, rats were administered D-Gal, followed 24 h later by a 2-hour session of UTOpiA treatment (Fig. S5A), at which time plasma AST and ALT levels were elevated. During the 3-day observation period, survival of UTOpiA-treated rats was significantly improved compared with the empty group (50% *vs.* 0%; Fig. S5B). D-gal-induced elevations in plasma AST, ALT and ammonia levels were significantly reduced after UTOpiA compared with empty treatment (Fig. S5C). Compared with rats in the empty group, UTOpiA-treated rats exhibited less hepatic injury, with reduced MPO-positive neutrophil accumulation and apoptosis (Fig. S5D–F). These data suggest that UTOpiA exhibits therapeutic efficacy in D-Gal-induced ALF by exerting anti-inflammatory effects.

### UTOpiA treatment activates the HNF4α transcriptional program in the liver of ACLF rats

**Fig. 4.**
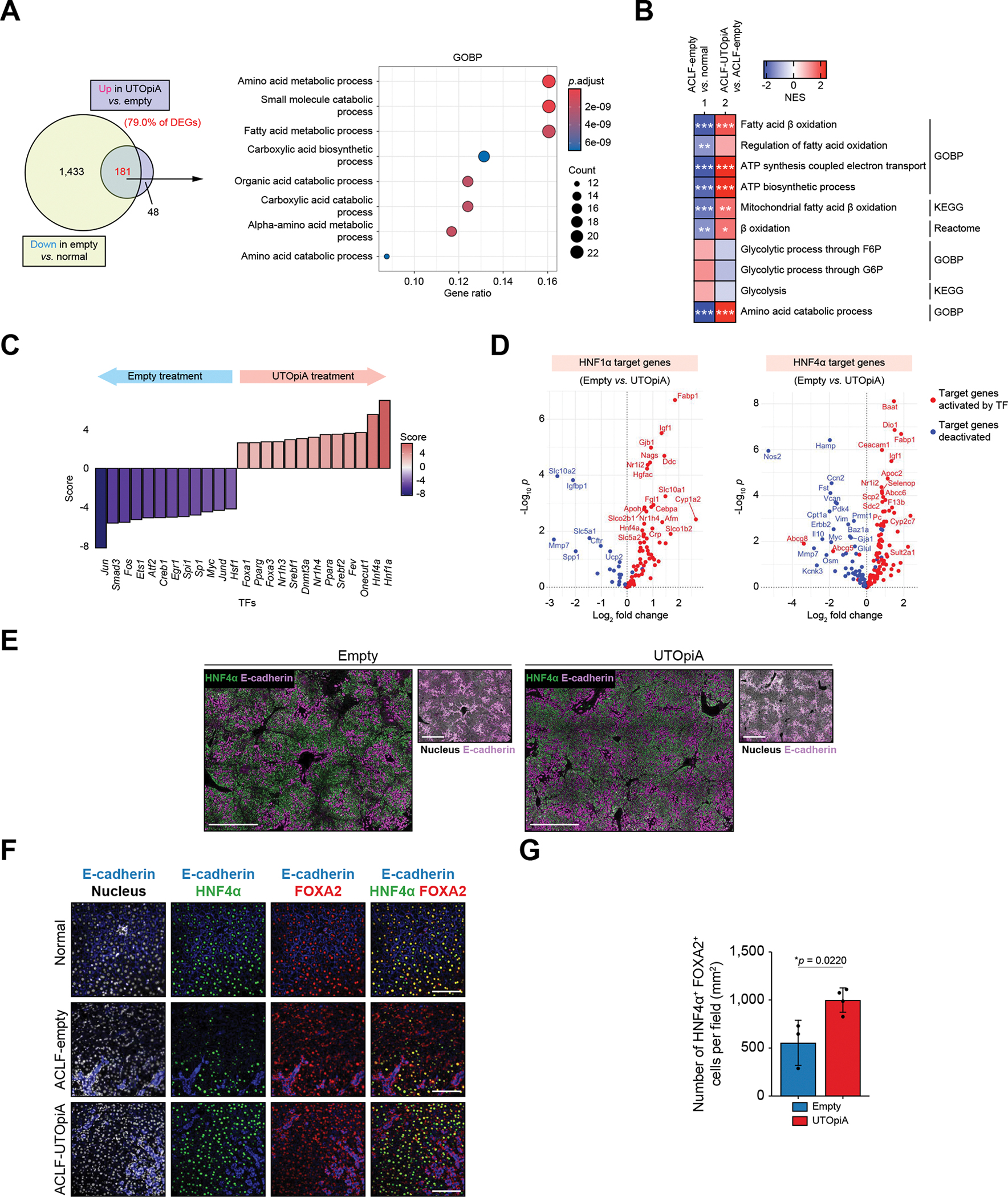
UTOpiA treatment activates HNF4α transcriptional program in livers of ACLF rats. (A) Venn diagrams showing the number of downregulated genes in liver tissues of BDL+LPS rats with empty treatment *vs.* normal rat, and of upregulated genes in those of BDL+LPS rats with UTOpiA treatment *vs.* with empty treatment. The overlapping regions indicate the number of DEGs reverted by UTOpiA treatment. GO analysis of biological processes for the reverted genes is also shown. (B) Gene set-enrichment analysis showing the differential pathways related to fatty acid oxidation, mitochondrial ATP, and glycolysis between ACLF empty column-treated and normal groups, as well as between empty column- and UTopiA-treated groups. *p <0.05; **p <0.01; ***p <0.001. (C) The activity changes of transcription factors between empty column- and UTopiA-treated groups inferred by RNA-seq data. (D) Volcano plots showing differential expression of HNF1α (left) and HNF4α (right) target genes between empty column- and UTOpiA-treated livers. Genes in red indicate activation by the transcription factor while those in blue are deactivated. (E) Immunofluorescence of the liver tissues in BDL+LPS rats with empty and UTOpiA treatments (24 h post LPS induction) to visualize nucleus (white), HNF4α (green) and E-cadherin (purple). Scale bar, 1 mm. (F,G) Immunofluorescence of the liver tissues in BDL+LPS rats with empty and UTOpiA treatments to visualize nucleus (white), HNF4α (green), FOXA2 (red) and E-cadherin (blue). Normal liver tissue was also included (F). Scale bar, 100 μm. Quantification of the number of cells positive for HNF4α and FOXA (G). Data are shown as mean ± SD (Student’s *t* test). ACLF, acute-on-chronic liver failure; BDL, bile duct ligation; DEGs, differentially expressed genes; GO, gene ontology; HNF1α, hepatocyte nuclear factor 1 alpha; RNA-seq, RNA-sequencing.

We next asked how UTOpiA influences the transcriptional states of hepatocytes in recipient rats. In our RNA-seq analysis of livers harvested 24 h post treatment, 79.0% of the DEGs upregulated by UTOpiA (181 genes) corresponded to genes that were downregulated in ACLF compared with normal livers (Fig. 4A). These genes were related to amino acid and fatty acid metabolism as revealed by gene ontology (Fig. 4A and S6A). Further, gene set-enrichment analysis showed upregulation of fatty acid oxidation and ATP synthesis in mitochondria and amino acid catabolism by UTOpiA, while glycolysis tended to be downregulated (Fig. 4B). The indicated mitochondrial dysfunction and amino acid-fueled metabolism are hallmarks associated with systemic inflammation in patients with ACLF.^34,35^ We next applied a prior knowledge-based statistical method, decoupleR,^36^ to our RNA-seq data to infer transcription factor activity following UTOpiA treatment. HNF1a and HNF4α were identified as the top activated transcription factors compared with the empty group (Fig. 4C,D). Both HNF1a and HNF4α establish the cross-regulatory network for downstream developmental and metabolic genes, with HNF4α serving as the central regulator of the hepatic transcriptional program.^37,38^ This was validated by enrichment analysis using chromatin immunoprecipitation-sequencing and transcription factor perturbation datasets, indicating higher HNF4α activity in the UTOpiA *vs*. the empty group (Fig. S6B and C). We subsequently performed immunofluorescence of HNF4α in ACLF rat liver tissue harvested 24 h post treatment. In the empty group, we found scattered parenchymal areas with negative or very weak positivity for HNF4α (Fig. 4E, left). In contrast, HNF4α was more uniformly expressed in livers of UTOpiA-treated rats (Fig. 4E, right). The cells negative for HNF4α observed in the empty group were unlikely to be necrotic since they retained intact nuclei and expressed FOXA2 (Fig. 4F). The number of cells positive for both HNF4α and FOXA2 was significantly increased in the UTOpiA group (Fig. 4F,G). These data suggest that the dysregulated expression of liver metabolic genes in ACLF was restored through the rewiring of HNF4α-dependent liver functional genes.

### UTOpiA treatment promotes liver regeneration responses in ACLF rats

**Fig. 5.**
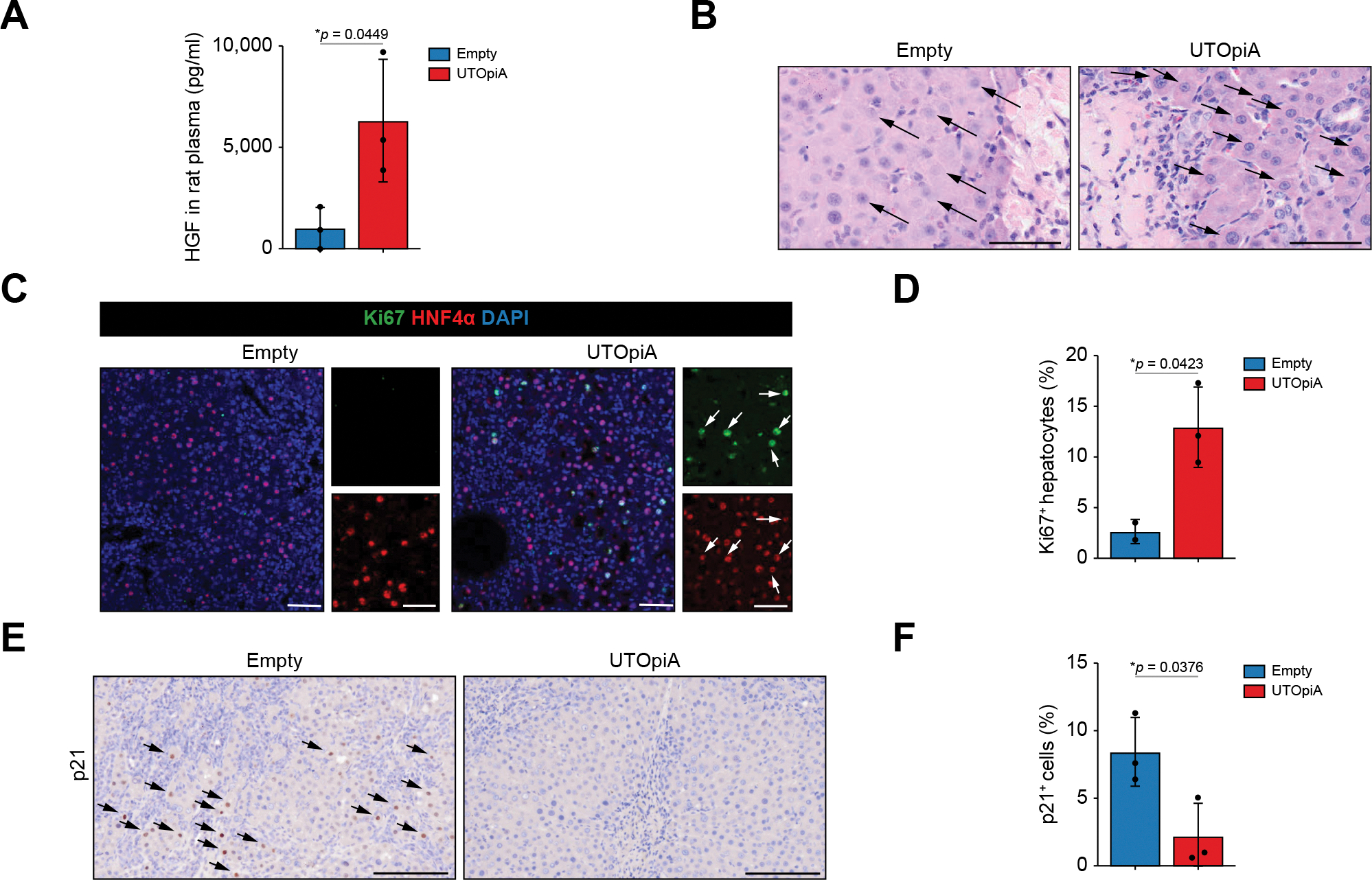
UTOpiA treatment promotes liver regeneration responses in ACLF rats. (A) Plasma levels of HGF in empty column- and UTOpiA-treated groups 2 h after the treatment. Data are shown as mean ± SD (Student’s *t* test). (B) Representative images of H&E staining of liver tissues in BDL+LPS rats with empty and UTOpiA treatments (72 h after LPS induction). In the empty treatment group, swollen hepatocytes were observed with the limited number of proliferating hepatocytes (arrows). In the UTOpiA treatment group, hepatocytes with normal morphology were occupied in the periphery of the injured areas (arrowheads). Scale bar, 50 μm. (C,D) Immunofluorescence of the liver tissues in BDL+LPS rats with empty and UTOpiA treatments (72 h post LPS induction) to visualize nucleus (blue), HNF4α (red) and Ki67 (green) (C). Scale bar, 50 μm (left), 150 μm (right). Quantification of the number of Ki67-positive hepatocytes (D). Data are shown as mean ± SD (Student’s *t* test). (E,F) Immunohistochemistry of p21 of the liver tissues in BDL+LPS rats with empty and UTOpiA treatments (24 h post LPS induction) (E). Bar, 100 μm. Quantification of the number of p21-positive cells (F). Data are shown as mean ± SD (Student’s *t* test). ACLF, acute-on-chronic liver failure; BDL, bile duct ligation; LPS, lipopolysaccharide.

Immediately after UTOpiA treatment, we observed increased plasma levels of HGF (Fig. 5A), which is known to trigger a proliferative response in hepatocytes during liver regeneration.^39^ As impaired hepatic regeneration is a key pathologic feature of ACLF, we next analyzed liver regenerative responses during the post-recovery phase. In the UTOpiA group, hepatocytes surrounding the necrotic area showed signs of recovery, characterized by increased cell density and dense nuclear staining at 72 h after LPS induction (Fig. 5B). Ki67 staining showed an increase in the number of proliferative cells in the UTOpiA group compared with the empty group (Fig. 5C, D). Most Ki67-positive cells expressed HNF4α, indicating an increase in hepatocyte proliferation. Hepatocytes positive for p21, a cell cycle inhibitor protein,^40^ accumulated in the livers of the empty group, which was evident 6 h after LPS induction. By contrast, the UTOpiA treatment led to a significant reduction in the number of p21-positive cells (Fig. 5E,F). Collectively, these data suggest that UTOpiA treatment promoted regnerative responses in hepatocytes of ACLF rats.

### AFP knockout of iHLCs compromises the therapeutic benefit of UTOpiA in ACLF rats

To determine if iHLC-secreted proteins play a role in the therapeutic benefit of UTOpiA treatment, we focused on AFP.^41,42^ Indeed, iHLCs derived from both WT and TKO iPSCs secreted more AFP into the extracellular milieu than PHHs and HepG2 cells *in vitro* (Fig. S7A). A human-specific ELISA showed that human AFP was detectable in rat plasma immediately after UTOpiA treatment at a level comparable to human albumin (Fig. S7B).

**Fig. 6.**
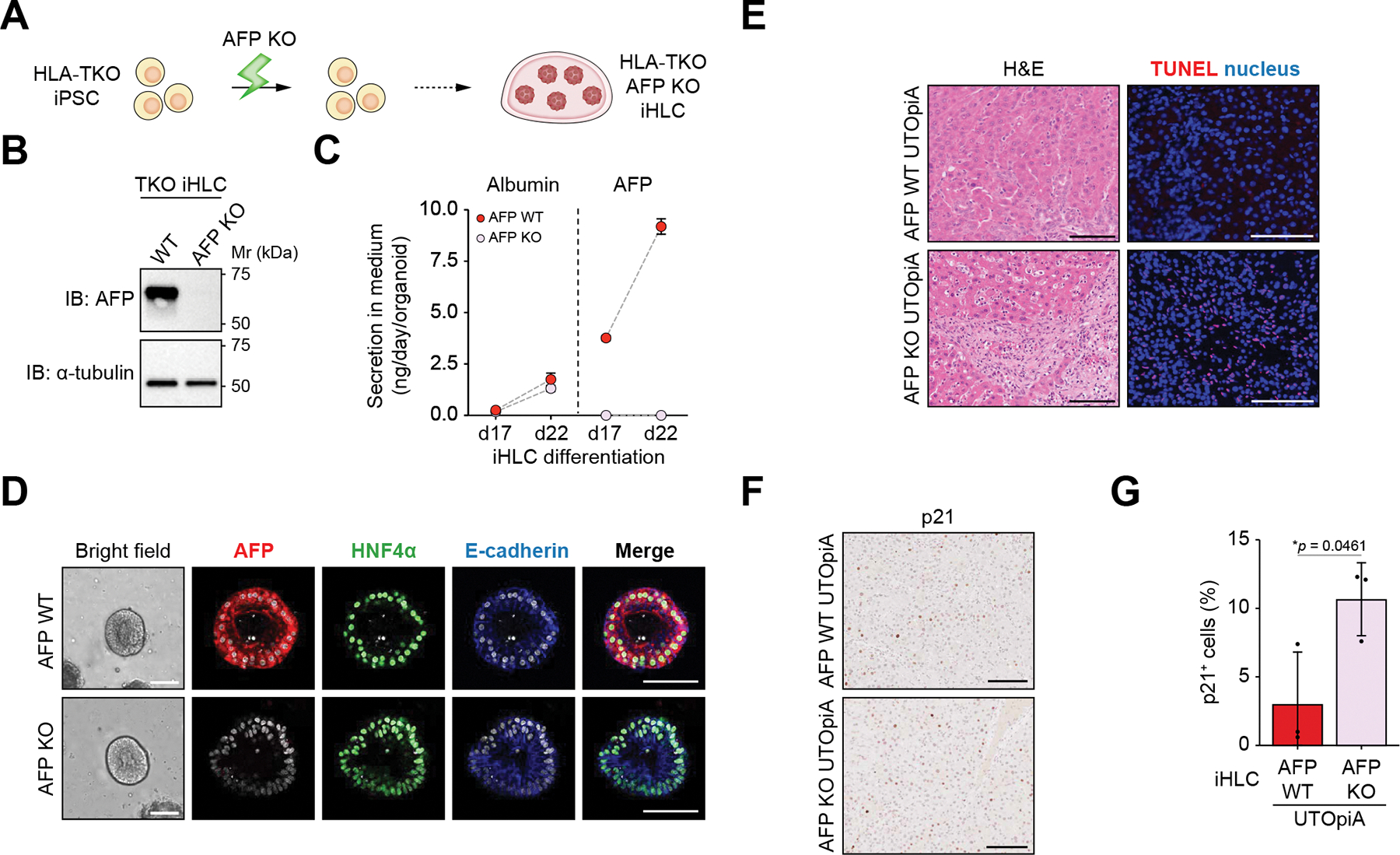
AFP knockout of iHLCs compromises the therapeutic benefit of UTOpiA in ACLF rats. (A) Schematic diagram of AFP knockout (KO) in iHLCs. (B) Immunoblotting showing AFP expression in WT and KO iHLCs. (C) Quantitative measurement of albumin and AFP secretion along iHLC differentiation by ELISA. (D) Whole-mount immunostaining of AFP WT and KO iHLCs to visualize AFP, E-cadherin and HNF4α. Scale bar, 100 μm. (E) Representative images of H&E and TUNEL staining of liver tissues (24 h after LPS induction) in BDL+LPS rats treated with UTOpiA using AFP WT or KO iHLCs. Scale bar, 100 μm. (F) Immunohistochemistry of p21 in liver tissues (24 h after LPS induction) in BDL+LPS rats treated with UTOpiA using AFP WT or KO iHLCs. Scale bar, 100 μm. (G) Quantification of the number of p21-positive cells. Data are shown as mean ± SD (Student’s *t* test). AFP, alpha-fetoprotein; ACLF, acute-on-chronic liver failure; BDL, bile duct ligation; iHLC, induced hepatocyte-like cell; KO, knockout; LPS, lipopolysaccharide; WT, wild-type.

To assess the contribution of iHLC-derived AFP to the therapeutic benefit of UTOpiA, we generated AFP HLA-TKO iPSCs via CRISPR-Cas9 editing (Fig. 6A and S7C). AFP deletion in the differentiated iHLCs was confirmed by qPCR, immunoblotting, immunofluorescence, and ELISA of culture supernatants (Fig. 6B–D and S7D). AFP-KO iHLCs showed no major differences from WT iHLCs in morphology, hepatic marker expression, or albumin secretion (Fig. 6C,D, and S7D). We then applied UTOpiA containing either AFP WT or KO iHLCs in ACLF rats. Rats treated with UTOpiA containing AFP-KO iHLCs exhibited greater hepatic injury 24 h post treatment, as assessed by TUNEL staining (Fig. 6E). Immunohistochemistry revealed that more p21-positive cells accumulated in livers of rats treated with AFP-KO iHLCs compared to those with AFP WT iHLCs (Fig. 6F,G). Ki67 staining showed no difference in hepatocyte proliferation between AFP WT and KO iHLC treatment (Fig. S7E and F). AFP-KO in iHLCs tended to compromise the survival benefit of UTOpiA treatment up to 72 h (Fig. S7G). Collectively, these data suggest that AFP secreted by iHLCs contributes to the therapeutic mechanism underlying protection against ACLF pathology.

## Discussion

ACLF is characterized by a systemic inflammatory response and rapid deterioration of the function of the liver and other vital organs, leading to mortality rates exceeding 50%.^4^ With the exception of liver transplantation, there are currently no definitive therapies that can recapitulate the central synthetic and metabolic roles of the liver. The UTOpiA BAL system, comprising an iHLC column downstream of a GMA column, alleviates the SIRS-like phenotype and provides metabolic and regenerative support, directly targeting key aspects of ACLF pathogenesis. By targeting acute inflammation and toxin accumulation – challenges poorly addressed by current intensive care – this whole blood extracorporeal circuit may improve patient outcomes.

SIRS in ACLF represents a state of complex immune dysfunction, characterized by persistent inflammation, cytokine overproduction, and multiorgan failure, leading to markedly increased mortality.^43^ A recent pilot study showed that GMA improved overall survival at 90 and 180 days in patients with corticosteroid-nonresponsive or -intolerant severe alcohol-related hepatitis.^6^ Whether this approach provides specific benefits in ACLF has not yet been evaluated. In our study, GMA significantly reduced hepatic neutrophil infiltration and systemic cytokines (*e.g*. IL-6, TNF-α, and IL-10), which affect the prognosis of ACLF,^44,45^ thus providing broad anti-inflammatory action beyond neutrophils and monocytes. GMA protects iHLCs during extracorporeal circulation by reducing direct contact with activated leukocytes, thereby permitting whole blood exposure to the cellular components.^46^ This configuration is a key differentiator from conventional BALs since our UTOpiA system requires no plasma separation device.

As allogeneic iPSC-derived hepatocytes and liver organoids face immune rejection due to HLA mismatch, recent research has focused on HLA gene knockout strategies to create hypoimmunogenic donor cells. The current state-of-the-art involves deleting key HLA class I and II genes via CRISPR-Cas9, for example, by the knockout of β2-microglobulin (B2M),^47^ and CIITA expression. Recent studies show that HLA knockout, achieved by disrupting B2M/CIITA, can generate hepatocyte-, endothelial-, and stellate-like lineages that function indistinguishably from wild-type cells.^48^ However, loss of all HLA-I triggers natural killer (NK) cell “missing-self” responses to trigger NK cell attack.^47^ To counter this, we focused on the selective removal of the most immunogenic class I loci (HLA-A and HLA-B) while sparing HLA-C, which can engage inhibitory KIR receptors on NK cells.^49^ These HLA-edited iPSCs can efficiently generate iHLCs comparable to unedited iPSCs which express albumin, uptake bile analogues, metabolize bilirubin, and produce urea, reflecting a mature hepatocyte phenotype. The developmental maturation of iPSCs into hepatocytes is not detectably impaired by deleting HLA-A, HLA-B, and B2M^48^ or CIITA. While alginate provides a physical barrier to iHLCs in UTOpiA, its semipermeable nature still permits the diffusion of immunogenic molecules; for example, membrane fragments released during necrosis may carry HLA, which stimulates alloreactive responses. This strategy of immune engineering preserves functionality and could facilitate off-the-shelf allogeneic iPSC-derived liver support in clinical settings.

The iHLC-based UTOpiA BAL system demonstrates robust therapeutic effects in preclinical models of ACLF and ALF through dual mechanisms of metabolic restoration and inflammatory resolution. Mechanistic evidence indicates that UTOpiA treatment enhances hepatocellular function, as evidenced by upregulation of the master hepatocyte transcription factor HNF4α, and concurrently suppresses systemic inflammatory mediators, with significant reductions in circulating pro-inflammatory cytokines such as TNF-α and IL-6. This combination of improved metabolic detoxification and dampened hyperinflammation translates into markedly better outcomes in animal models, including significant mortality benefits. Notably, UTOpiA’s integrated approach contrasts with the ELAD system, which relies on an immortalized HepG2 C3A hepatoblastoma cell line for partial detoxification and showed limited efficacy in ACLF, failing to improve 3-month survival in severe alcohol-related hepatitis. This lack of efficacy in ACLF was further confirmed in our study using a HepG2 C3A cell-incorporated extracorporeal device. In contrast, UTOpiA integrates detoxification, regeneration, and inflammation control in a single platform, achieved by only ~300 million iHLCs, far fewer than the >10 billion hepatocytes required by other systems.^50,51^ By simultaneously supporting liver metabolic capacity and tapering the inflammatory cascade, UTOpiA addresses key drivers of ACLF pathogenesis, underscoring a significant advance over prior BAL therapies.

We observed AFP as a key factor secreted from iHLCs that contributes to the therapeutic benefits of the UTOpiA system. When AFP-KO iHLCs were used, more p21-positive cells accumulated compared with AFP WT iHLC treatment, indicating a reduced regenerative response. Emerging evidence suggests that AFP may have active biological roles in adult liver pathology. For instance, AFP knockdown in hepatocellular carcinoma cells has been shown to induce G0/G1 cell cycle arrest via upregulation of p21 and downregulation of cyclin D1 and CDK4, highlighting a potential role in promoting cell proliferation,^52^ which mirrors our observation that AFP-KO iHLCs failed to suppress p21 expression *in vivo*. AFP also contributes to immune modulation during fetal development and tumor progression.^53^ Such immune regulatory properties could be relevant in the inflammatory context of ACLF, where tissue repair must proceed alongside resolution of systemic inflammation. Additionally, AFP expression has been shown to be transiently re-induced during liver regeneration following acute injury, further supporting a role beyond that of a passive lineage marker.^54^ Further investigations will be required to delineate the molecular functions of AFP, exogenously delivered from fetal liver-like hepatocytes, in conditions of liver failure.

Thus, the combined use of GMA and iHLCs in UTOpiA holds promise for addressing the multifactorial pathology of ACLF by targeting both immune dysregulation and hepatic insufficiency. However, important limitations must be acknowledged. The rat BDL+LPS model used here, while useful for proof of concept, does not capture the full heterogeneity of human ACLF. Moreover, several unknowns remain, including potential immunogenicity or migration of the iHLCs, the feasibility of repeated treatment sessions, and the scalability of sufficient iHLC manufacturing to support widespread clinical use. Finally, large animal studies are needed to confirm human physiological relevance and safety in a setting more comparable to human anatomy and immunology. Addressing these issues through further preclinical research and engineering refinements will be crucial for translating this promising GMA-iHLC tandem approach into a safe, scalable, and effective therapy for severe liver failure.

## Supplementary Material

Suppl 2

Suppl 1

Suppl 3

Supplementary data

Supplementary data to this article can be found online at https://doi.org/10.1016/j.jhep.2025.08.038.

## Data Availability

The RNA-seq data reported in this paper has been deposited to NCBI Gene Expression Omnibus (GEO) under the accession number GSE 299117.

## Highlights

- HLA-A, HLA-B, and CIITA triple knockout iPSCs can generate iHLCs with metabolic and detoxification functions.
- UTOpiA combines iHLCs and GMA to rescue preclinical models of ACLF and ALF.
- UTOpiA suppresses neutrophil infiltration and systemic inflammation.
- AFP secretion from iHLCs promotes liver regeneration in ACLF rats.
- The tandem GMA-iHLC circuit enables whole blood bioartificial liver therapy.

## Impact and implications

Restoring hepatic metabolism while controlling inflammation in acutely decompensated cirrhosis remains a major unmet clinical need. By combining HLA-modified human induced pluripotent stem cell–derived liver organoids with granulocyte and monocyte apheresis, we developed a bioartificial liver system that manages inflammation, restores liver function, and promotes regeneration in two rat models of severe liver failure. This therapy could offer an off-the-shelf treatment option for patients with life-threatening liver failure.

## Abbreviations

ACLF: acute-on-chronic liver failure
ALF: acute liver failure
ALT: alanine aminotransferase
AST: aspartate aminotransferase
BAL: bioartificial liver
BDL: bile duct ligation
FG: foregut
GMA: granulocyte and monocyte apheresis
iHLC: induced hepatocyte-like cell
iPSC: human induced pluripotent stem cell
LPS: lipopolysaccharide
MASLD: metabolic dysfunction-associated steatotic liver disease
PHHs: primary human hepatocytes
qPCR: quantitative PCR
SIRS: systemic inflammatory response syndrome
TKO: triple knockout
WT: wild-type

## References

[1] Moreau R, Jalan R, Gines P, Acute-on-chronic liver failure is a distinct syndrome that develops in patients with acute decompensation of cirrhosis. Gastroenterology 2013;144:1426–1437. 1437.e1421–1429. 23474284 10.1053/j.gastro.2013.02.042

[2] European association for the study of the EASL clinical practice guidelines on acute-on-chronic liver failure. J Hepatol 2023;79:461–491. 37364789 10.1016/j.jhep.2023.04.021

[3] Mezzano G, Juanola A, Cardenas A, Global burden of disease: acute-on-chronic liver failure, a systematic review and meta-analysis. Gut 2022;71:148–155. 33436495 10.1136/gutjnl-2020-322161

[4] Liu LX, Zhang Y, Nie Y, Assessing the prediction effect of various prognosis model for 28-day mortality in acute-on-chronic liver failure patients. Risk Manag Healthc Pol 2020;13:3155–3163. 10.2147/RMHP.S281999 PMC7778450 33402854

[5] Sundaram V, Shah P, Wong RJ, Patients with acute on chronic liver failure grade 3 have greater 14-day waitlist mortality than status-1a patients. Hepatology 2019;70:334–345. 30908660 10.1002/hep.30624 PMC6597310

[6] Kasuga R, Chu PS, Taniki N, Granulocyte-monocyte/macrophage apheresis for steroid-nonresponsive or steroid-intolerant severe alcohol-associated hepatitis: a pilot study. Hepatol Commun 2024;8. 10.1097/HC9.0000000000000371 PMC10830070 38285891

[7] Chen HS, Joo DJ, Shaheen M, Randomized trial of spheroid reservoir bioartificial liver in porcine model of posthepatectomy liver failure. Hepatology 2019;69:329–342. 30022502 10.1002/hep.30184 PMC6527364

[8] Carpentier B, Gautier A, Legallais C. Artificial and bioartificial liver devices: present and future. Gut 2009;58:1690–1702. 19923348 10.1136/gut.2008.175380

[9] Thompson J, Jones N, Al-Khafaji A, Extracorporeal cellular therapy (ELAD) in severe alcoholic hepatitis: a multinational, prospective, controlled, randomized trial. Liver Transpl 2018;24:380–393. 29171941 10.1002/lt.24986 PMC5873437

[10] Shi XL, Gao Y, Yan Y, Improved survival of porcine acute liver failure by a bioartificial liver device implanted with induced human functional hepatocytes. Cell Res 2016;26:206–216. 26768767 10.1038/cr.2016.6 PMC4746613

[11] Wang Y, Zheng Q, Sun Z, Reversal of liver failure using a bioartificial liver device implanted with clinical-grade human-induced hepatocytes. Cell Stem Cell 2023;30:617–631.e618. 37059100 10.1016/j.stem.2023.03.013

[12] Li WJ, Zhu XJ, Yuan TJ, An extracorporeal bioartificial liver embedded with 3D-layered human liver progenitor-like cells relieves acute liver failure in pigs. Sci Transl Med 2020;12. 10.1126/scitranslmed.aba5146 32641490

[13] Chen S, Wang J, Ren H, Hepatic spheroids derived from human induced pluripotent stem cells in bio-artificial liver rescue porcine acute liver failure. Cell Res 2020;30:95–97. 31827240 10.1038/s41422-019-0261-5 PMC6951340

[14] Shinozawa T, Kimura M, Cai Y, High-fidelity drug-induced liver injury screen using human pluripotent stem cell-derived organoids. Gastroenterology 2021;160:831–846.e810. 33039464 10.1053/j.gastro.2020.10.002 PMC7878295

[15] Kimura M, Iguchi T, Iwasawa K, En masse organoid phenotyping informs metabolic-associated genetic susceptibility to NASH. Cell 2022;185:4216–4232.e4216. 36240780 10.1016/j.cell.2022.09.031 PMC9617783

[16] Ouchi R, Togo S, Kimura M, Modeling steatohepatitis in humans with pluripotent stem cell-derived organoids. Cell Metab 2019;30:374–384.e376. 31155493 10.1016/j.cmet.2019.05.007 PMC6687537

[17] Reza HA, Farooqui Z, Reza AA, Synthetic augmentation of bilirubin metabolism in human pluripotent stem cell-derived liver organoids. Stem Cell Rep 2023;18:2071–2083. 10.1016/j.stemcr.2023.09.006 PMC10679658 37832542

[18] Xu H, Wang B, Ono M, Targeted disruption of HLA genes via CRISPR-cas9 generates iPSCs with enhanced immune compatibility. Cell Stem Cell 2019;24:566–578.e567. 30853558 10.1016/j.stem.2019.02.005

[19] Engelmann C, Sheikh M, Sharma S, Toll-like receptor 4 is a therapeutic target for prevention and treatment of liver failure. J Hepatol 2020;73:102–112. 31987990 10.1016/j.jhep.2020.01.011

[20] Kitano Y, Nishimura S, Kato TM, Generation of hypoimmunogenic induced pluripotent stem cells by CRISPR-Cas9 system and detailed evaluation for clinical application. Mol Ther Methods Clin Dev 2022;26:15–25. 35755947 10.1016/j.omtm.2022.05.010 PMC9198376

[21] Tripathi DM, Vilaseca M, Lafoz E, Simvastatin prevents progression of acute on chronic liver failure in rats with cirrhosis and portal hypertension. Gastroenterology 2018;155:1564–1577. 30055171 10.1053/j.gastro.2018.07.022

[22] Ma S, Xu Q, Deng B, Granulocyte and monocyte adsorptive apheresis ameliorates sepsis in rats. Intensive Care Med Exp 2017;5:18. 28342161 10.1186/s40635-017-0129-2 PMC5366986

[23] Hu X, White K, Olroyd AG, Hypoimmune induced pluripotent stem cells survive long term in fully immunocompetent, allogeneic rhesus macaques. Nat Biotechnol 2024;42:413–423. 37156915 10.1038/s41587-023-01784-x PMC10940156

[24] Deuse T, Tediashvili G, Hu X, Hypoimmune induced pluripotent stem cell-derived cell therapeutics treat cardiovascular and pulmonary diseases in immunocompetent allogeneic mice. Proc Natl Acad Sci U S A 2021;118. 10.1073/pnas.2022091118 PMC8285900 34244428

[25] Gravina A, Tediashvili G, Zheng Y, Synthetic immune checkpoint engagers protect HLA-deficient iPSCs and derivatives from innate immune cell cytotoxicity. Cell Stem Cell 2023;30:1538–1548.e1534. 37922880 10.1016/j.stem.2023.10.003

[26] Ho BX, Teo AKK, Ng NHJ. Innovations in bio-engineering and cell-based approaches to address immunological challenges in islet transplantation. Front Immunol 2024;15:1375177. 38650946 10.3389/fimmu.2024.1375177 PMC11033429

[27] Mavri-Damelin D, Eaton S, Damelin LH, Ornithine transcarbamylase and arginase I deficiency are responsible for diminished urea cycle function in the human hepatoblastoma cell line HepG2. Int J Biochem Cell Biol 2007;39:555–564. 17098461 10.1016/j.biocel.2006.10.007

[28] Kayumov M, Habimana R, Kim D, Extracorporeal circulation models in small animals: beyond the limits of preclinical research. Acute Crit Care 2023;38:1–7. 36935529 10.4266/acc.2023.00381 PMC10030238

[29] Lopez-Mendez TB, Santos-Vizcaino E, Pedraz JL, Cell microencapsulation technologies for sustained drug delivery: clinical trials and companies. Drug Discov Today 2021;26:852–861. 33242694 10.1016/j.drudis.2020.11.019

[30] Pais-Roldán P, Edlow BL, Jiang Y, Multimodal assessment of recovery from coma in a rat model of diffuse brainstem tegmentum injury. Neuroimage 2019;189:615–630. 30708105 10.1016/j.neuroimage.2019.01.060 PMC6642798

[31] Mogahed E, El-Karaksy H, Zaki H, Autoimmune hepatitis in Egyptian children: a single center experience. Int J Immunopathol Pharmacol 2022;36. 20587384211073265. 35231187 10.1177/20587384211073265 PMC8894955

[32] Tecchio C, Micheletti A, Cassatella MA. Neutrophil-derived cytokines: facts beyond expression. Front Immunol 2014;5:508. 25374568 10.3389/fimmu.2014.00508 PMC4204637

[33] Horn KJ, Fulte S, Yang M, Neutrophil responsiveness to IL-10 impairs clearance of Streptococcus pneumoniae from the lungs. J Leukoc Biol 2024;115:4–15. 37381945 10.1093/jleuko/qiad070 PMC10768920

[34] Zaccherini G, Aguilar F, Caraceni P, Assessing the role of amino acids in systemic inflammation and organ failure in patients with ACLF. J Hepatol 2021;74:1117–1131. 33276029 10.1016/j.jhep.2020.11.035

[35] Zhang IW, Curto A, Lopez-Vicario C, Mitochondrial dysfunction governs immunometabolism in leukocytes of patients with acute-on-chronic liver failure. J Hepatol 2022;76:93–106. 34450236 10.1016/j.jhep.2021.08.009

[36] Badia IMP, Velez Santiago J, Braunger J, decoupleR: ensemble of computational methods to infer biological activities from omics data. Bioinform Adv 2022;2:vbac016. 36699385 10.1093/bioadv/vbac016 PMC9710656

[37] Lau HH, Ng NHJ, Loo LSW, The molecular functions of hepatocyte nuclear factors - in and beyond the liver. J Hepatol 2018;68:1033–1048. 29175243 10.1016/j.jhep.2017.11.026

[38] Odom DT, Zizlsperger N, Gordon DB, Control of pancreas and liver gene expression by HNF transcription factors. Science 2004;303:1378–1381. 14988562 10.1126/science.1089769 PMC3012624

[39] Michalopoulos GK. Hepatostat: liver regeneration and normal liver tissue maintenance. Hepatology 2017;65:1384–1392. 27997988 10.1002/hep.28988

[40] Chang BD, Watanabe K, Broude EV, Effects of p21Waf1/Cip1/Sdi1 on cellular gene expression: implications for carcinogenesis, senescence, and age-related diseases. Proc Natl Acad Sci U S A 2000;97:4291–4296. 10760295 10.1073/pnas.97.8.4291 PMC18232

[41] Pak VN. The use of alpha-fetoprotein for the treatment of autoimmune diseases and cancer. Ther Deliv 2018;9:37–46. 29216804 10.4155/tde-2017-0073

[42] Munson PV, Adamik J, Butterfield LH. Immunomodulatory impact of alpha-fetoprotein. Trends Immunol 2022;43:438–448. 35550875 10.1016/j.it.2022.04.001

[43] Engelmann C, Habtesion A, Hassan M, Combination of G-CSF and a TLR4 inhibitor reduce inflammation and promote regeneration in a mouse model of ACLF. J Hepatol 2022;77:1325–1338. 35843375 10.1016/j.jhep.2022.07.006

[44] Solé C, Solà E, Morales-Ruiz M, Characterization of inflammatory response in acute-on-chronic liver failure and relationship with prognosis. Sci Rep 2016;6:32341. 27578545 10.1038/srep32341 PMC5006032

[45] Liu L, Xiao N, Chen P, IL-10 predicts the prognosis of patients with hepatitis B virus-related acute-on-chronic liver failure combined with spontaneous bacterial peritonitis. Front Med 2023;10:1188300. 10.3389/fmed.2023.1188300 PMC10562642 37822472

[46] Damania A, Hassan M, Shirakigawa N, Alleviating liver failure conditions using an integrated hybrid cryogel based cellular bioreactor as a bioartificial liver support. Sci Rep 2017;7:40323. 28079174 10.1038/srep40323 PMC5227920

[47] Koga K, Wang B, Kaneko S. Current status and future perspectives of HLA-edited induced pluripotent stem cells. Inflamm Regen 2020;40:23. 33014207 10.1186/s41232-020-00132-9 PMC7528263

[48] Trionfini P, Romano E, Varinelli M, Hypoimmunogenic human pluripotent stem cells as a powerful tool for liver regenerative medicine. Int J Mol Sci 2023;24. 10.3390/ijms241411810 PMC10380710 37511568

[49] Xu H, Wang B, Ono M, Targeted disruption of HLA genes via CRISPR-cas9 generates iPSCs with enhanced immune compatibility. Cell Stem Cell 2019;24:566–578 e567. 30853558 10.1016/j.stem.2019.02.005

[50] Felgendreff P, Tharwat M, Hosseiniasl SM, Preclinical experience of the mayo spheroid reservoir bioartificial liver (SRBAL) in management of acute liver failure. Livers 2022;2:387–399.

[51] Dixit V, Gitnick G. Artificial liver support: state of the art. Scand J Gastroenterol 1996;31:101–114. 10.3109/00365529609094760 8898446

[52] Lin Z, Pan R, Wu L, AFP-HSP90 mediated MYC/MET activation promotes tumor progression in hepatocellular carcinoma and gastric cancers. Cancer Cell Int 2024;24:283. 39135041 10.1186/s12935-024-03455-6 PMC11321088

[53] Lu Y, Lin B, Li M. The role of alpha-fetoprotein in the tumor microenvironment of hepatocellular carcinoma. Front Oncol 2024;14:1363695. 38660138 10.3389/fonc.2024.1363695 PMC11039944

[54] Ben-Moshe S, Veg T, Manco R, The spatiotemporal program of zonal liver regeneration following acute injury. Cell Stem Cell 2022;29:973–989.e910. 35659879 10.1016/j.stem.2022.04.008

