# Supplementary material for: Reversal of ACLF and ALF using whole blood extracorporeal system combining HLA-depleted liver organoids with granulocyte-monocyte apheresis
Source: J Hepatol. Author manuscript; Available in PMC 2026 Jan 5. (PMC12765605; doi:10.1016/j.jhep.2025.08.038)
Supplement: Suppl 2 [file NIHMS2123507-supplement-Suppl_2.docx]

**Journal of Hepatology**

**CTAT methods**

Tables for a “Complete, Transparent, Accurate and Timely account” (CTAT) are now mandatory for all revised submissions. The aim is to enhance the reproducibility of methods.

- Only include the parts relevant to your study
- Refer to the CTAT in the main text as ‘Supplementary CTAT Table’
- Do not add subheadings
- Add as many rows as needed to include all information
- Only include one item per row

**If the CTAT form is not relevant to your study, please outline the reasons why:**

|  |
| --- |

- 1. **Antibodies**

| **Name** | **Citation** | **Supplier** | **Cat no.** | **Clone no.** |
| --- | --- | --- | --- | --- |
| E-cadherin |  | R&D Systems | AF648 |  |
| HNF-4α |  | abcam | ab92378 | EPR3648 |
| HNF4α |  | Perseus Proteomics | PP-K9218-00 | K9218 |
| HNF4α |  | Invitrogen | MA5-44801 | JE63-17 |
| Albumin |  | Bethyl Laboratories | A80-129A |  |
| ZO1 |  | Cell Signaling Technology | 13663 | D6L1E |
| AFP |  | Santa Cruz | sc-8399 | C3 |
| α-tubulin |  | Cell Signaling Technology | 3873 | DM1A |
| MPO |  | abcam | ab9535 |  |
| Cleaved caspase-3 |  | Cell Signaling Technology | 9661S |  |
| Ki67 |  | abcam | ab16667 | SP6 |
| FOXA2 |  | Abnova | H00003170-M01 | 7E6 |
| p21 |  | Invitrogen | MA5-42680 | 4Z1H9 |
| HLA-A-A24 (Alexa Fluor 647) |  | MBL | K0208-A64 | 17A10 |
| HLA B7 |  | Bio-Rad Laboratories | MCA986 | BB7.1 |

- 1. **Cell lines**

| **Name** | **Citation** | **Supplier** | **Cat no.** | **Passage no.** | **Authentication test method** |
| --- | --- | --- | --- | --- | --- |
| HepG2/C3A |  | ATCC | CRL-3581 | P1-P10 | Morphology check by microscope |
| Ff-XT28s05_cont (human iPSC) | PMID: [30853558](https://www.ncbi.nlm.nih.gov/pubmed/30853558) | CiRA Foundation |  | P14-24 | STR analysis  Morphology check by microscope |
| Ff-XT28s05-Abo_To #14-4 (human iPSC) | PMID: [30853558](https://www.ncbi.nlm.nih.gov/pubmed/30853558) | CiRA Foundation |  | P29-P39 | STR analysis  Morphology check by microscope |
| Ff-I01s04-AbII KO-54 (human iPSC) |  | CiRA Foundation |  | P35-P45 | STR analysis  Morphology check by microscope |

- 1. **Organisms**

| **Name** | **Citation** | **Supplier** | **Strain** | **Sex** | **Age** | **Overall n number** |
| --- | --- | --- | --- | --- | --- | --- |
| Rat |  | Sankyo Laboratory | SD | Male | 8-11 weeks | 60 |
| Rat |  | CLEA Japan | SD | Male | 8-11 weeks | 20 |

- 1. **Sequence based reagents**

| **Name** | **Sequence** | **Supplier** |
| --- | --- | --- |
| ACTB-F | AAGGAGATCACTGCCCTGGCACCC | Invitrogen |
| ACTB-R | AAGCCATGCCAATCTCATCTTG | Invitrogen |
| POU5F1-F | GGTGGAGGAAGCTGACAACA | Invitrogen |
| POU5F1-R | CTGATCTGCTGCAGTGTGGG | Invitrogen |
| NANOG-F | CATAAATCTAGAGACTCCAGG | Invitrogen |
| NANOG-R | AGGACCTCCAGAAGGAAAAG | Invitrogen |
| HNF4A-F | GAGCGATCCAGGGAAGATCA | Invitrogen |
| HNF4A-R | CATACTGGCGGTCGTTGATG | Invitrogen |
| CEBPA-F | AGAAGTCGGTGGACAAGAACAGCA | Invitrogen |
| CEBPA-R | ATTGTCACTGGTCAGCTCCAGCA | Invitrogen |
| ALB-F | CATCTCAGCCTACCATGAGAATAA | Invitrogen |
| ALB-R | TAGACAGGGTGTTGGCTTTAC | Invitrogen |
| ABCC2-F | TGTCGAATGGCAGATGTGTC | Integrated DNA Technologies |
| ABCC2-R | CTTCACCTCCATTACCCTCTTC | Integrated DNA Technologies |
| ABCB11-F | TCTGATCTCTAAGCCACTGAATG | Integrated DNA Technologies |
| ABCB11-R | GAAATGACAGCTCTGGTAGGAC | Integrated DNA Technologies |
| ARG1-F | TGGCAGATATACAGGGAGTCA | Integrated DNA Technologies |
| ARG1-R | ACTCCACTGACAACCACAAG | Integrated DNA Technologies |
| ASL-F | GGTAATAGGCAAGGTCAGTGG | Integrated DNA Technologies |
| ASL-R | GAAGCTGTGTTTGAAGTGTCAG | Integrated DNA Technologies |
| ASS1-F | CTGACATCCTCAATGAACACCT | Integrated DNA Technologies |
| ASS1-R | GGCTGAAGGAACAAGGCTAT | Integrated DNA Technologies |
| CPS1-F | GAATCTGGCCTCCAACTGAT | Integrated DNA Technologies |
| CPS1-R | GTGAGCACAGACTTTGATGAGT | Integrated DNA Technologies |
| OTC-F | GGACGATTCTATGCCCTTGA | Integrated DNA Technologies |
| OTC-R | TATTACCTTTGCTCCCTCACTG | Integrated DNA Technologies |
| ARG2-F | CATCAACCCAGACAACACAAAG | Integrated DNA Technologies |
| ARG2-R | GTTAGCAGAGCTGTGTCAGAT | Integrated DNA Technologies |
| AFP-F | GCTGACCTGGCTACCATATTT | Invitrogen |
| AFP-R | TGTTCATCTCCAGTGGGTTTC | Invitrogen |
| CYP3A4-F | GCTGAGGATGAAGAATGGAAGA | Invitrogen |
| CYP3A4-R | CTCCATACTGGGCAATGATAGG | Invitrogen |
| sgAFP-T | CACCGAACTTATCTCTGCAGTACAT | Invitrogen |
| sgAFP-B | AAACATGTACTGCAGAGATAAGTTC | Invitrogen |

- 1. **Biological samples**

| **Description** | **Source** | **Identifier** |
| --- | --- | --- |
| Cryo Human Hepatocytes Induction Qualified | Lonza | HUCPG |

- 1. **Deposited data**

| **Name of repository** | **Identifier** | **Link** |
| --- | --- | --- |
|  |  |  |

- 1. **Software**

| **Software name** | **Manufacturer** | **Version** |
| --- | --- | --- |
| Graphpad Prism | Graphpad Software | 10.4.1 |
| FlowJo | BD Biosciences | 10.10.0 |
| Fiji | PMID: 22743772 | 2.14.0 |
| R | <https://www.R-project.org> | 4.3.2 |
| RStudio | Poist Software | 2023.12.1+402 |

- 1. **Other (e.g. drugs, proteins, vectors etc.)**

| **Compounds** | **Supplier** | **Cat no.** |
| --- | --- | --- |
| Lipopolysaccharide | Sigma-Aldrich | L2630-25MG |
| D-galactosamine | Fujifilm Wako Pure Chemical Corporation | 079-02054 |
| CLF | Corning | 451041 |
|  |  |  |
| **Chemicals, peptides, and recombinant proteins for cell culture** | **Supplier** | **Cat no.** |
| MEM Non-essential amino acid solution | Thermo Fisher Scientific | 11140-050 |
| Human Activin A | R&D Systems | 338-AC-500 |
| Human BMP4 | R&D Systems | 314-BP-050 |
| B27 | Gibco | 17504-044 |
| Fetal bovine serum | Thermo Fisher Scientific | SH30071.03 |
| B27 | Thermo Fisher Scientific | 17504-044 |
| N2 | Thermo Fisher Scientific | 17502-048 |
| HEPES | Thermo Fisher Scientific | 15630-080 |
| GlutaMAX | Thermo Fisher Scientific | 35050-061 |
| FGF4 | PEPROTECH | 100-31 |
| CHIR99021 | R&D Systems | 4423 |
| Retinoic acid | Sigma-Aldrich | R2625 |
| Human HGF | PeproTech | 100-39 |
| Dexamethasone | Sigma-Aldrich | D4902 |
| Human Oncostatin M | PeproTech | 300-10 |
| DAPT | nacalai tesque | 18767-14 |
| EGF | R&D | 236-EG-200 |
| A83-01 | tocris | 2939/10 |
| VEGF | Life Tech | PHC9391 |
| bFGF | nacalai tesque | 19155-81 |
| IGF1 | Peprotech | AF-100-11-100µg |
| Ascorbic acid | nacalai tesque | 13048-42 |
| Y-27632 | nacalai tesque | 18188-04 |
| IFN-γ | Proteintech | HZ-1301 |
| sodium pyruvate | Gibco | 11360070 |
| Pen/Strep | Gibco | 15140-122 |
| Matrigel | Corning | 356237 |
| TrypLE Express | Gibco | 12605-010 |
| Cell Recovery Solution | Corning | 354253 |
| BSA fraction V | Gibco | 15260-037 |
| Sodium Alginate | Fujifilm Wako Pure Chemical Corporation | 194-13325 |
| CELL BANKER 1 | Nippon Zenyaku Kogyo Co, Ltd | CB011 |
| Cell Recovery Solution | Corning | 354253 |
| iMatrix-511 | Nippi | 892012 |
| Accutase | nacalai tesque | 12679-54 |
| TRI reagent | Molecular Research Center, Inc. | TR118 |
| RNA later Stabilization Solution | Invitrogen | AM7020 |
|  |  |  |
| **Medium** | **Supplier** | **Cat no.** |
| StemFit AK02N | Ajinomoto Co | AK02N |
| Hepatocyte Culture Medium BulletKit | Lonza | CC-3198 |
| Advanced DMEM/F12 | Thermo Fisher Scientific Inc. | 12634010 |
| RPMI 1640 | nacalai tesque | 30264-56 (10 botles) |
| HCM Complete Medium | Lonza | CC-3199 plus CC-4182 |
| DMEM (low glucose) | nacalai tesque | 08456-65 |
|  |  |  |
| **Commercial Assays** | **Supplier** | **Cat no.** |
| Human Albumin ELISA Kit | Bethyl Laboratories, Inc. | E88-129 |
| AFP Human ELlSA Kit | RayBiotech | ELH-AFP-1 |
| Mouse/Rat HGF Quantikine ELISA kit | R&D Systems | MHG00 |
| IL-6 Rat ELISA Kit | R&D Systems | R6000B |
| Rat TNF alpha ELISA kit | abcam | ab236712 |
| QuantiChrom Urea Assay Kit | BioAssay Systems | DIUR-100 |
| Glucose-Glo-Assay | Promega | J6021 |
| DRI-CHEM NX500V | Fujifilm CO. |  |
| LIVE/DEAD Fixable Near-IR Dead Cell Stain Kit | Invitrogen | L10119 |
| FastGene RNA Premium Kit | NIPPON Genetics, Co., Ltd. | FG-80050 |
| Cell Staining buffer | BioLegend | 420201 |
| Ghost Dye Violet 450 | TONBO | 13-0863-T100 |
| Cell Lysis Buffer | Cell signaling | 9803 |
| Protein Assay BCA Kit | nacalai tesque | 06385-00 |
| Clarity Western ECL Substrate | Bio-Rad | 1705060 |
| TUNEL Assay Kit | Cell Signaling Technology | 48513 |
|  |  |  |
| **Vector** |  |  |
| pSpCas9(BB)-2A-GFP (PX458) | Addgene | 48138 |
|  |  |  |
| **Other** |  |  |
| EZSPHERE SP MICROPLATE 6 Well with Lid | IWAKI | 4810-900-SP |
| 96-well ultra-low attachment plate | Corning | 3474 |
| 6-well ultra-low attachment plate | ThermoFisher Scientific Inc. | 174932 |
| Mobicol Classic Column (1 ml), with 2 Different Screw Cap, without Filter | MoBiTec Molecular Biotechnology | M1002 |
| Mobicol Filter, small, Pore Size 90 μm | MoBiTec Molecular Biotechnology | M2190 |
| Adacolumn | JIMRO |  |

- 1. **Please provide the details of the corresponding methods author for the manuscript:**

| **Takanori Takebe**, Graduate School of Medicine and Premium Research Institute for Human Metaverse Medicine (WPI-PRIMe), The University of Osaka, Suita, Osaka 565-0871, Japan  Phone: +81 (6) 6879 3860, Email: [Takanori.Takebe@cchmc.org](mailto:Takanori.Takebe@cchmc.org) |
| --- |

**2.0 Please confirm for randomised controlled trials all versions of the clinical protocol are included in the submission. These will be published online as supplementary information.**

|  |
| --- |
