# Supplementary material for: Reversal of ACLF and ALF using whole blood extracorporeal system combining HLA-depleted liver organoids with granulocyte-monocyte apheresis
Source: J Hepatol. Author manuscript; Available in PMC 2026 Jan 5. (PMC12765605; doi:10.1016/j.jhep.2025.08.038)
Supplement: Suppl 1 [file NIHMS2123507-supplement-Suppl_1.pdf]

Reversal of ACLF and ALF using whole blood extracorporeal system combining HLA-depleted liver organoids with granulocyte-monocyte apheresis

Hitomi Yamaguchi, Yosuke Yoneyama, Kentaro Ichimura, Kanae Ohtsu, Mika Soen, Chiharu Moriya, Maki Kumagai, Rob Myers, G Mani Subramanian, Takanori Takebe

Table of contents

Supplementary methods……………………………………………………………………...2

Supplementary figures…………………………………………………………………......4

Supplementary tables…………………………………………………………….………14

Supplementary references……………………………………………………….………15

Supplementary methods

Encapsulation of iHLC into alginate

Sodium alginate powder (Wako Pure Chemicals, Cat # 194-13325) was dissolved in water to a concentration of 1.5% (w/v) and filtered through a 0.22 μm filter to sterilize. iHLCs were collected and centrifuged at 300 × g for 5 min at 4°C. The supernatant was removed and the iHLC pellet was crushed by gently tapping the tube. iHLCs were resuspended in alginate solution at a density of approximately 4 × 10^3 organoids per 300 μl. Droplets of alginate containing iHLCs (approximately 30 μl per droplet) were dropped directly into a 0.4% BaCl2 solution and polymerized at room temperature for 15 min. Polymerization was allowed to occur. The microcapsules were washed three times with PBS to remove excess Ba^{2+} ions. The encapsulated iHLCs were maintained in HCM medium until used for BAL experiments. For PHH and HepG2, cells were made into spheroids as described above and then encapsulated. They were resuspended in alginate solution at a concentration of approximately 4 × 10^3 spheroids per 300 μl. Droplets of alginate (approximately 30 μl per droplet) were dropped directly into 0.4% $BaCl_2$ solution and allowed to polymerise for 15 min at room temperature. The microcapsules were washed three times with PBS to remove excess Ba^{2+} ions.

Induction of acute liver failure in rats

In order to induce acute liver failure without pre-existing chronic liver injury, D-galactosamine was injected i.p. at a dose of 2,000 mg/kg diluted in saline solution in adult male Sprague-Dawley rats (weight 300-350 g) while optimized with reference to the previous studies[1, 2]. Since D-galactosamine-induced ALF was severe and quickly lethal for rats, we chose to perform the extracorporeal circulation 24 h after the administration of D-galactosamine. We set 96 h after the extracorporeal circulation as an endpoint in this experiment.

CRISPR-Cas9-mediated knockout of AFP in human iPSC

We chose the Ff-I01s04-AbII TKO human iPSC as a parental cell line to further knock out *AFP* gene. The guide RNA sequence targeting AFP was chosen to avoid off-targeting and locate in the downstream of and proximity to start codon-containing exon of *AFP* gene by using CRISPick tool[3, 4], and subcloned

into pX458 vector (Addgene plasmid # 48138). The oligonucleotides for the sgRNA subcloning were as follows: top 5'-CACCGAACTTATCTCTGCAGTACAT-3', bottom 5'-AAACATGTACTGCAGAGATAAGTTC-3'. The insert sequence of the resulting pX458-sgAFP was verified by Sanger sequencing. For gene targeting, 10 μg of pX458-sgAFP was transfected into 1×10^6 cells of human iPSC, followed by plating under feeder-free conditions for 48 h in AK02N medium containing 10 μM Y-27632. The transfected cells were subjected to cell sorting on a BD FACSAria III to enrich GFP-positive cells. We also isolated GFP-negative cells for deriving non-targeting control cell lines. We validated the specific targeting of the isolated GFP-positive fraction by using Guide-it Indel Identification Kit (TaKaRa) with the following primers to amplify the exon 2 of *AFP* gene: forward 5'-TGGGATATGAATGGCAAACC-3' and reverse 5'-GGAATCAAAAGGTCAATGTCG-3'. The single cell-derived clones were subsequently obtained from the GFP-positive fraction by limiting dilution. The isolated clones were genotyped by PCR of exon 2 with the primers mentioned above and Sanger sequencing. The clone with biallelic nonsense mutations were further expanded and differentiated for subsequent experiments.

Immunoblotting

Total cell lysates were prepared using Cell Lysis Buffer (Cell Signaling Technology) containing Protease/Phosphatase Inhibitor Cocktail (Cell Signaling Technology) and protein concentrations were determined using a Protein Assay BCA Kit (nacalai tesque). For western blotting, protein samples were separated by sodium dodecyl sulfate-polyacrylamide gel electrophoresis and transferred to PVDF membranes (Bio-Rad). The membranes were blocked in Blocking One (nacalai tesque) at room temperature for 30 min. The membranes were then incubated with the following primary antibodies over night at 4°C: anti-AFP (#sc-8399m Santa Cruz) and anti-α-tubulin (#3873, Cell Signaling Technology). The membranes were subsequently incubated with horseradish peroxidase-conjugated goat anti-rabbit IgG and anti-mouse IgG (Bio-Rad) and visualized by using Clarity Western ECL Substrate (Bio-Rad).

Supplementary figures

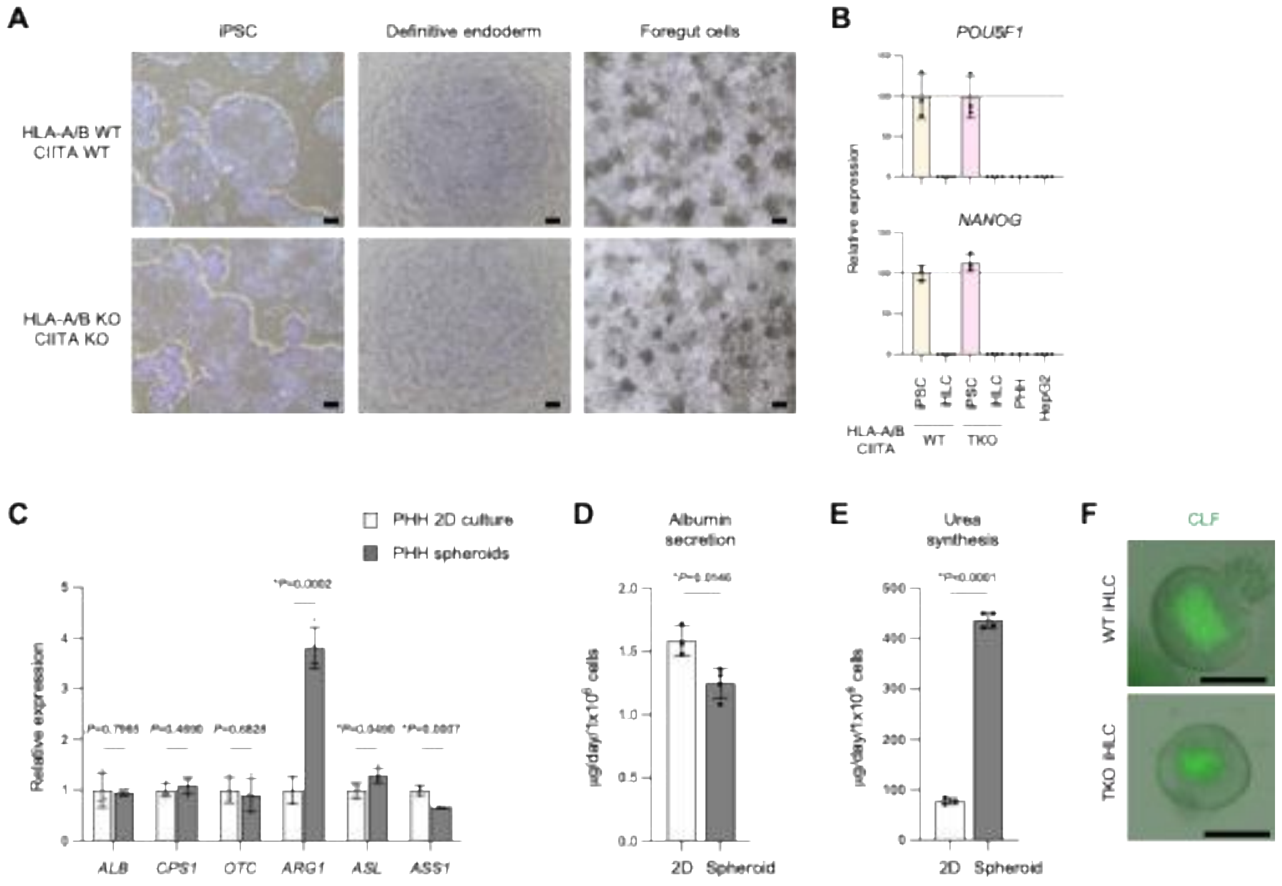

Fig. S1. Induction of definitive endoderm and foregut cells from HLA-A, B, CIITA knockout iPSCs.

(A) Phase contrast images of iPSCs, definitive endoderm, and foregut cells derived from WT and HLA-TKO iPSC lines. Scale bar, 100 μm.

(B) qRT-PCR analysis of pluripotency markers *POU5F1*and *NANOG* in WT and TKO iPSCs, iHLCs, PHH, and HepG2 spheroids.

(C) RT-qPCR analysis of genes related to urea cycle (*CPS1*, *OTC*, *ARG1*, *ASL*, *ASS1*) and albumin (*ALB*) in PHH monolayer culture (2D) and spheroids. Data are shown as mean ± SD folded by the values of the 2D culture (Student's *t*-test).

(D) Quantitative analysis of albumin secretion of PHH 2D culture and spheroids. Data are shown as mean ± SD (Student's *t*-test).

(E) Measurement of urea synthesis in PHH 2D culture and spheroids. Data are shown as mean ± SD (Student's *t*-test).

(F) Fluorescence image of CLF uptake in WT and TKO iHLCs. Scale bar 100 μm.

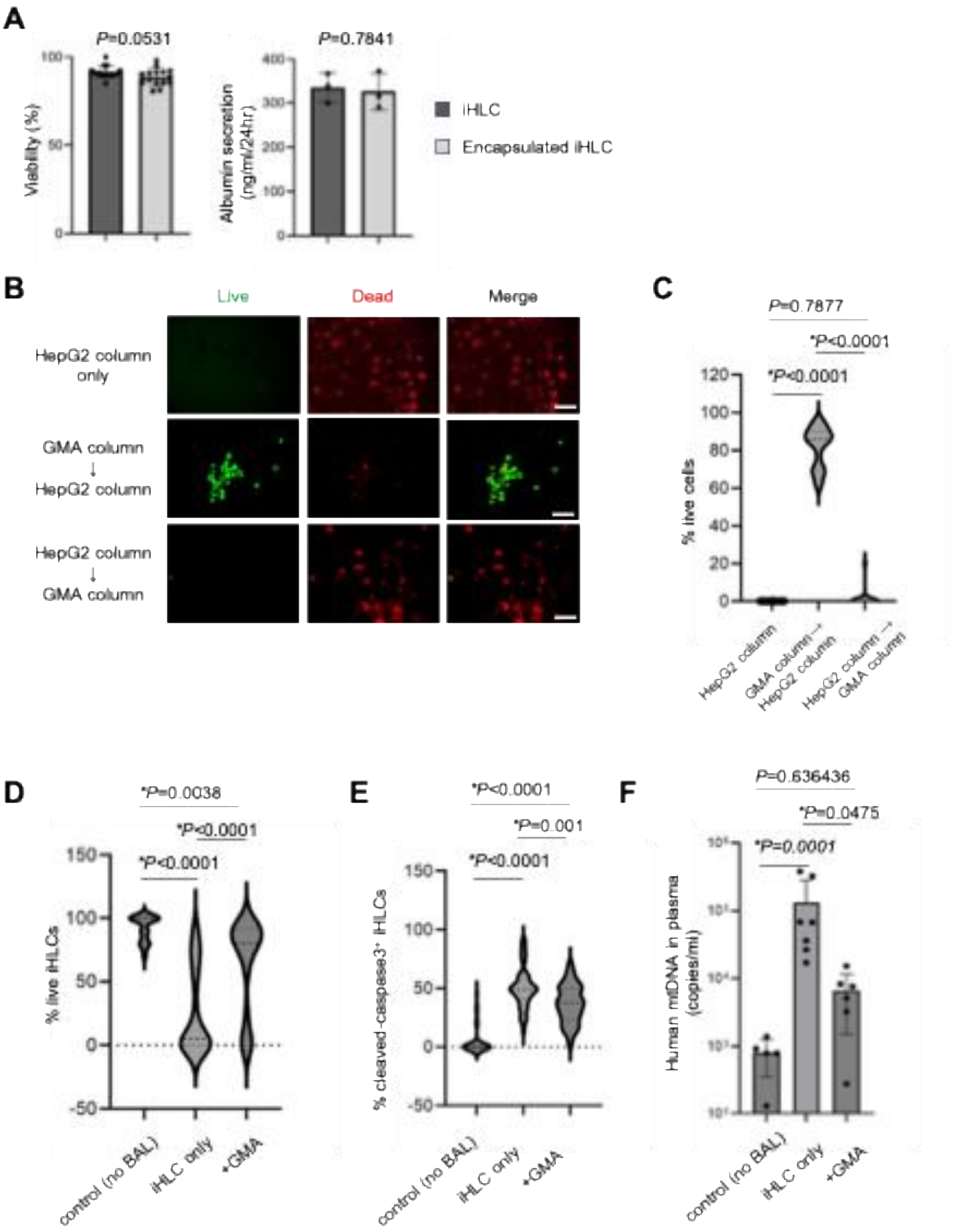

Fig. S2. Tandem connection with GMA improves the survival of iHLC

(A) Viability and albumin secretion level of alginate-encapsulated iHLCs. Viability of iHLCs by calcein-AM (green)/EthD-1 (red) staining. Albumin secretion level was determined by ELISA of the supernatant obtained from 24 hr culture *in vitro*. iHLCs cultured in Matrigel before encapsulation were set as a control. Data are shown as average ± SD (Student's *t*-test).

(B,C) Representative images of encapsulated HepG2 spheroids housed in the column of extracorporeal circulation with healthy rats (B). The GMA column was connected in tandem with two different configurations: GMA column -> HepG2 column and HepG2 column -> GMA column. Two hours after extracorporeal circulation, HepG2 spheroids were collected followed by staining with calcein-AM/EthD-1. Bar, 500 μm. The violin plot shows the percentage of live cells (C) (Tukey's post-hoc test).

(D) Quantification of the percentage of calcein-positive live iHLCs after extracorporeal circulation with or without GMA tandem connection in the ACLF rat model. After the treatment, iHLCs were collected from the column and stained with calcein-AM and EthD-1. Encapsulated iHLC without extracorporeal circulation was used as a control (Tukey's post-hoc test).

(E) Quantification of the percentage of cleaved caspase-3-positive iHLCs after extracorporeal circulation in the ACLF rat model. After the treatment, iHLCs were collected from the column followed by whole mount staining of cleaved caspase-3 and F-actin (Tukey's post-hoc test).

(F) Quantification of cell-free human mitochondrial DNA (mtDNA) detected in the plasma of ACLF model rats that received the extracorporeal circulation treatment. Cell-free DNA was extracted from rat plasma collected after the extracorporeal circulation. Quantitative PCR (qPCR) using human-specific mitochondrial DNA was performed. The standard for human mtDNA copy number was prepared by performing qPCR of both mtDNA and genomic DNA of iHLCs. Data are shown as average ± SD (Dunn's post-hoc test).

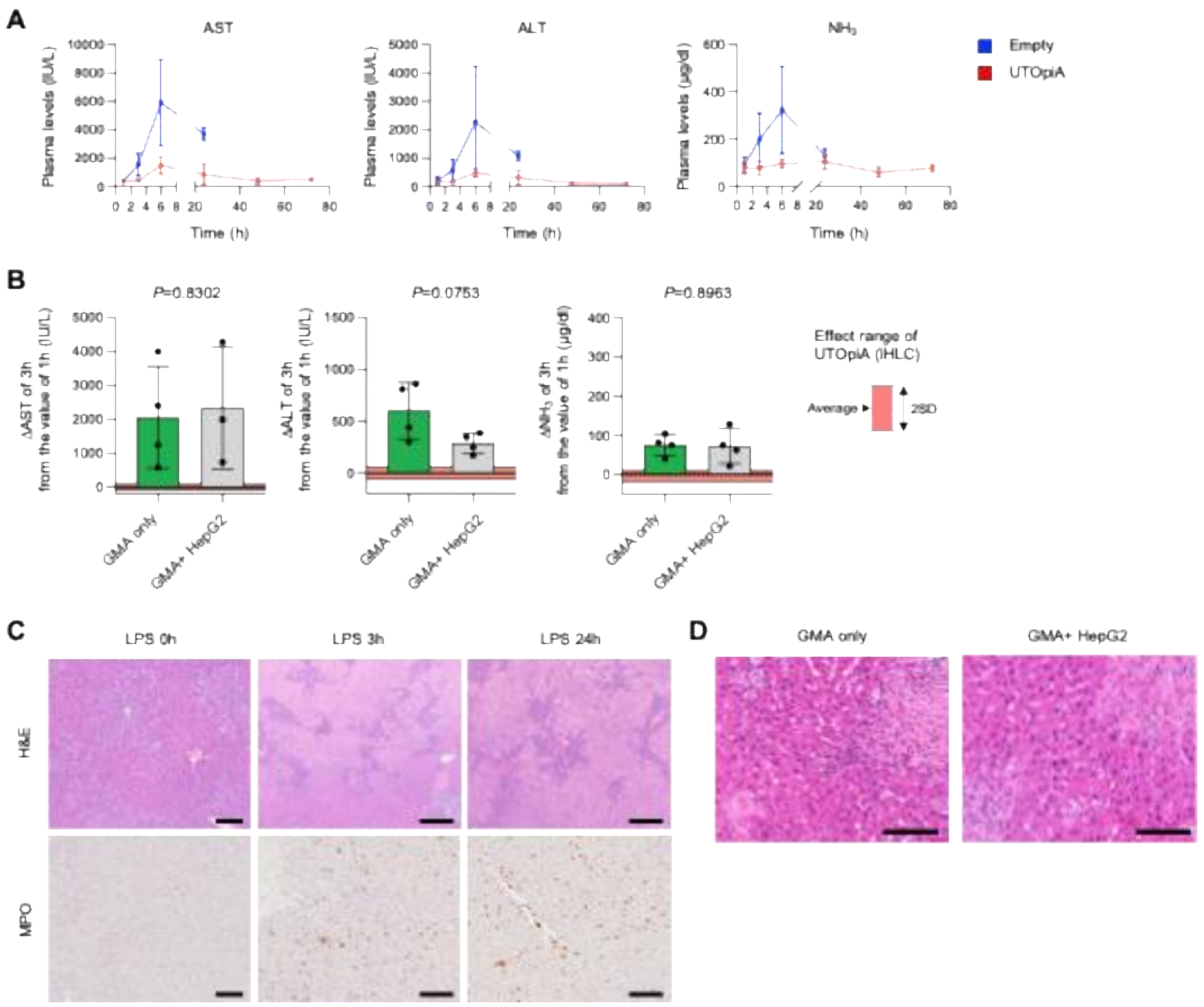

Fig. S3. Effects of UTOpiA on liver injury markers

(A) The plasma levels of AST, ALT, and ammonia in empty- and UTOpiA-treated rats. Data are shown as average ± SD.

(B) Relative changes in plasma levels of AST, ALT, and ammonia at the end of treatment compared to those at 1 hours in BDL+LPS rats treated with GMA only or GMA+HepG2. The values of differences before and after the treatment are shown as average ± SD (Student's *t*-test). The value ranges in pink were 2SD of effects by UTOpiA.

(C) Representative images of H&E and MPO staining of liver tissues in BDL rats with LPS administration. Scale bars, 200 μm (H&E) and 100 μm (MPO).

(D) Representative images of H&E staining of liver tissues (24 hours after treatment) in the BDL+LPS rats in GMA only and GMA+HepG2 groups. Scale bar, 100 μm.

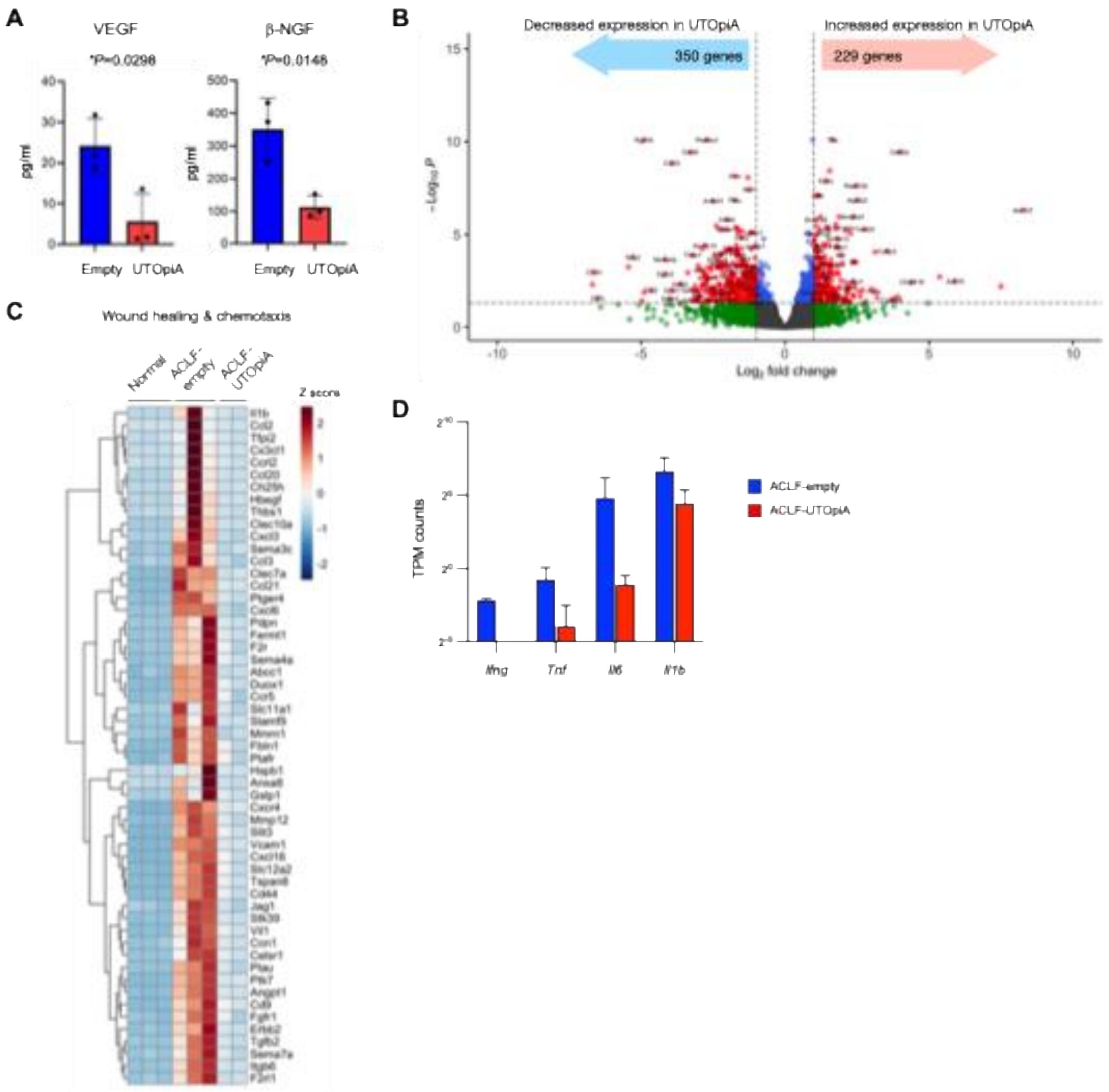

Fig. S4. Effect of UTOpiA treatment on inflammation markers

(A) Plasma level of VEGF and β-NGF in empty column- and UTOpiA-treated groups determined by cytokine array (Student's *t*-test).

(B) Volcano plot identifying genes that are differentially expressed (cutoff padj <0.05, |fold change| > 2) in rat liver tissues between empty-treated and UTOpiA-treated groups.

(C) The expression of genes related to wound healing and chemotaxis in normal liver, empty column- and UTOpiA-treated groups based on RNA-seq data.

(D) The gene expression of inflammatory cytokines *Ifng*, *Tnf*, *Il6*, and *Il1b* in rat liver of empty column- and UTOpiA-treated groups based on RNA-seq data.

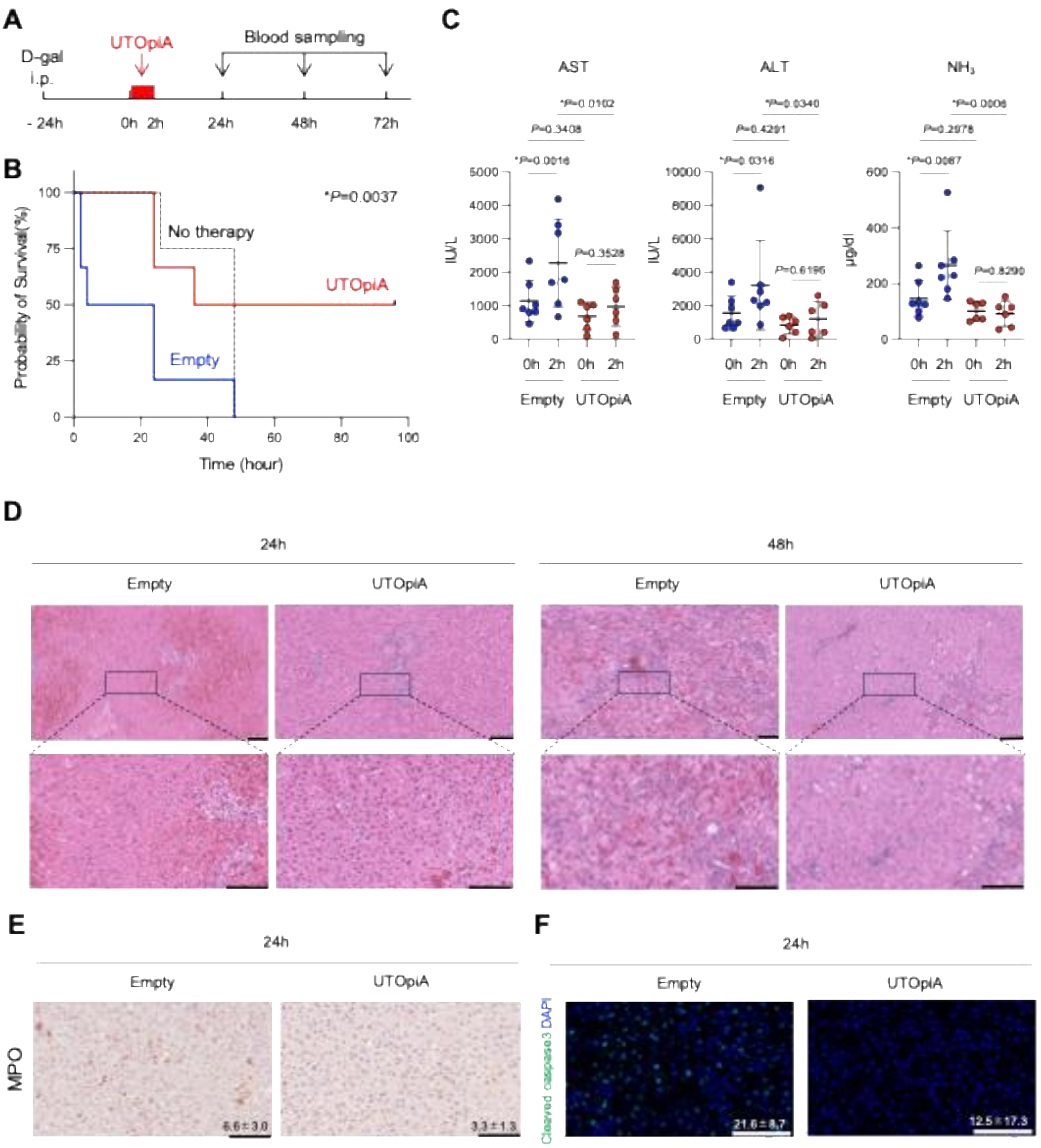

Fig. S5. UTOpiA rescues rats from D-galactosamine-induced ALF.

(A) Schematic diagram of the treatment protocol. 24 hours after intraperitoneal D-Gal injection into rats, the treatment of UTOpiA was performed for 2 hours.

(B) Kaplan-Meier survival curve of D-Gal rats treated with no therapy (n=4), empty column (n=6), and UTOpiA (n=6). *P=0.0037, log-rank (Mantel-Cox) test.

(C) Changes in plasma levels of AST, ALT, ammonia and total bilirubin (T-bil) at the end of the BAL treatment compared to those at the beginning (Fisher's LSD test).

(D) Representative images of H&E staining of liver tissues in the D-Gal-induced ALF rats in empty column- and UTOpiA- treated groups (24 and 48 hours after treatment). Scale bar, 100 μm.

(E) Immunohistochemistry of MPO-positive neutrophil in livers with empty or UTOpiA treatment (24 hours after treatment). Scale bar, 100 μm. The percentage of cells positive for MPO were indicated above the scale bar.

(F) Representative immunofluorescence images of cleaved caspase-3 in livers with empty or UTOpiA treatment (24 hours after treatment). Scale bar, 100 μm. The percentage of cells positive for cleaved caspase-3 were indicated above the scale bar.

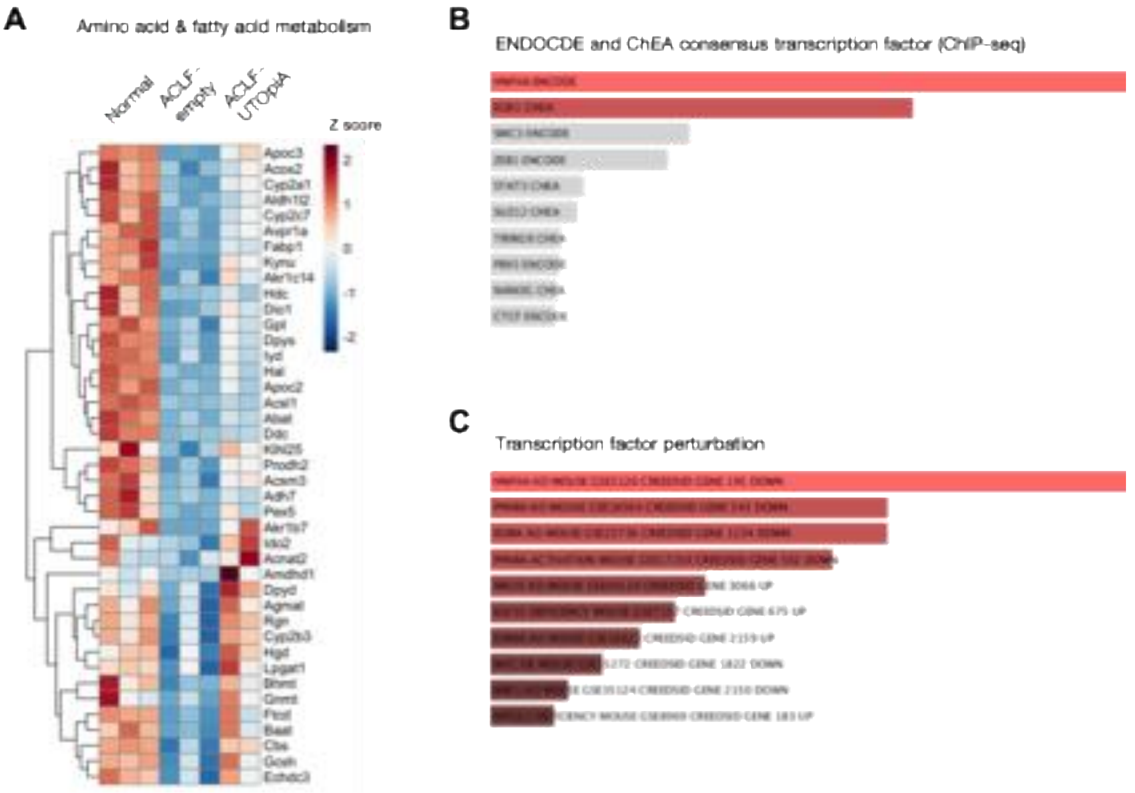

Fig. S6. RNA-seq analysis of rat liver tissues treated with UTOpiA

(A) The expression of genes related to amino acid and fatty acid metabolism in normal liver, empty column-and UTOpiA treated groups based on RNA-seq data.

(B) Enrichr analysis of genes that are upregulated as reverted differentially expressed genes using ChIP-seq dataset composed of ENCODE and ChEA consensus transcription factor.

(C) Enrichr analysis of genes that are upregulated as reverted differentially expressed genes using transcription factor perturbation dataset.

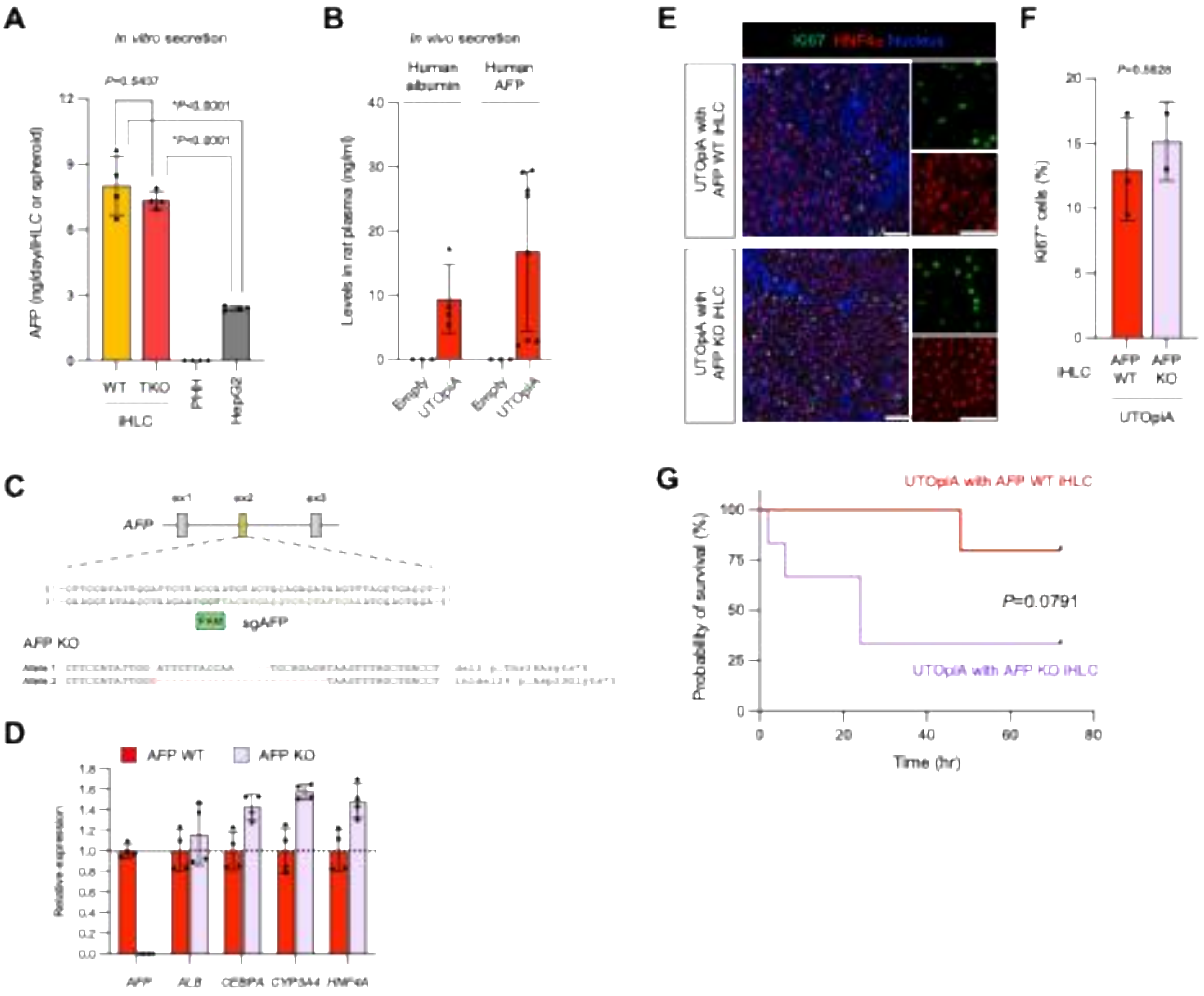

Fig. S7. AFP knockout in HLA-A, B, CIITA triple knockout iHLCs

(A) Quantitative analysis of AFP by ELISA measurement of culture supernatants *in vitro*. PHH and HepG2 spheroids were added as a control. Data are shown as mean ± SD with *P* values of the indicated comparisons (Tukey's post-hoc test).

(B) Plasma levels of human albumin and AFP in rats 2 hours after empty or UTOpiA treatment. Data are shown as mean ± SD.

(C) Schematic of CRISPR-Cas9 targeting for *AFP* gene (top). The obtained knockout clone was validated by Sanger sequencing of *AFP* exon 2, showing the premature termination in both alleles (bottom).

(D) RT-qPCR analysis of genes related to hepatocyte markers in AFP WT and KO iHLCs. Data are shown as mean ± SD folded by the values of AFP WT cells.

(E) Immunofluorescence of Ki67, HNF4α, and nucleus of the liver tissues in BDL+LPS rats treated with AFP WT or KO iHLC-implemented UTOpiA (72 hours after LPS induction). Scale bar, 50 μm.

(F) Quantification of the number of Ki67-positive cells. Data are shown as mean ± SD (Student's *t*-test).

(G) Kaplan-Meier survival curve of BDL+LPS rats treated with AFP WT (n=5) or KO (n=6) iHLC-implemented UTOpiA. *P*=0.0791, log-rank (Mantel-Cox) test.

Supplementary tables

Table S1. List of antibodies used in this study

**Primary antibodies**				
**Antibody**	**Host**	**Manufacturer**	**Catalog #**	**Dilution**
E-cadherin	Goat	R&D	AF648	1:500
HNF4α	Rabbit	abcam	ab92378	1:200
HNF4α	Rabbit	Invitrogen	MA5-44801	1:500
Albumin	Goat	Bethyl Laboratories	A80-129A	1:250
ZO1	Rabbit	Cell Signaling Technology	13663	1:200
AFP	Mouse	Santa Cruz	sc-8399	1:100
α-tubulin	Mouse	Cell Signaling Technology	3873	1:2000
MPO	Rabbit	abcam	Ab9535	1:50
Cleaved caspase-3	Rabbit	Cell Signaling Technology	9661S	1:400
Ki67	Rabbit	abcam	Ab16667	1:200
HNF4α	Mouse	Perseus Proteomics	PP-K9218-00	1:100
p21	Rabbit	Invitrogen	MA5-42680	1:100
FOXA2	Mouse	Abnova	H00003170-M01	1:100
HLA-A-A24	Mouse	MBL	K0208-A64	1:100
HLA B7	Mouse	Bio-Rad Laboratories	MCA986	1:100

**Secondary antibodies**			
**Antibody**	**Manufacturer**	**Catalog #**	**Dilution**
Donkey anti-mouse IgG, Alexa Fluor 647	Invitrogen	31571	1:1,1000
Donkey anti-Rabbit IgG, Alexa Fluor 555	Invitrogen	31572	1:1,1000
Donkey anti-Rabbit IgG, Alexa Fluor 647	Invitrogen	31573	1:1,1000
Donkey anti-Goat IgG, Alexa Fluor 488	Invitrogen	11055	1:1,1000

Table S2. List of RT-qPCR primers used in this study

Gene	Forward primer (5' to 3')	Reverse primer (5' to 3')
POU5F1	GGTGGAGGAAGCTGACAACA	CTGATCTGCTGCAGTGTGGG
NANOG	CATAAATCTAGAGACTCCAGG	AGGACCTCCAGAAGGAAAAG
ALB	CATCTCAGCCTACCATGAGAATAA	TAGACAGGGTGTTGGCTTTAC
CYP3A4	GCTGAGGATGAAGAATGGAAGA	CTCCATACTGGGCAATGATAGG
CEBPA	AGAAGTCGGTGGACAAGAACAGCA	ATTGTCACTGGTCAGCTCCAGCA
AFP	GCTGACCTGGCTACCATATTT	TGTTCATCTCCAGTGGGTTTC
HNF4A	GAGCGATCCAGGGAAGATCA	CATACTGGCGGTCGTTGATG
ABCC2	TGTCGAATGGCAGATGTGTC	CTTCACCTCCATTACCCTCTTC
ABCB11	TCTGATCTCTAAGCCACTGAATG	GAAATGACAGCTCTGGTAGGAC
ARG1	TGGCAGATATACAGGGAGTCA	ACTCCACTGACAACCACAAG
ASL	GGTAATAGGCAAGGTCAGTGG	GAAGCTGTGTTTGAAGTGTCAG
ASS1	CTGACATCCTCAATGAACACCT	GGCTGAAGGAACAAGGCTAT
CPS1	GAATCTGGCCTCCAACTGAT	GTGAGCACAGACTTTGATGAGT
OTC	GGACGATTCTATGCCCTTGA	TATTACCTTTGCTCCCTCACTG
ARG2	CATCAACCCAGACAACACAAAG	GTTAGCAGAGCTGTGTCAGAT

Supplementary references

[1] Jitraruch S, Dhawan A, Hughes RD, et al. Alginate microencapsulated hepatocytes optimised for transplantation in acute liver failure. PLoS One 2014;9:e113609.
[2] Ito T, Ishigami M, Matsushita Y, et al. Secreted ectodomain of SIGLEC-9 and MCP-1 synergistically improve acute liver failure in rats by altering macrophage polarity. Scientific reports 2017;7:44043.
[3] Doench JG, Fusi N, Sullender M, et al. Optimized sgRNA design to maximize activity and minimize off-target effects of CRISPR-Cas9. Nat Biotechnol 2016;34:184-191.
[4] Sanson KR, Hanna RE, Hegde M, et al. Optimized libraries for CRISPR-Cas9 genetic screens with multiple modalities. Nat Commun 2018;9:5416.
